# Supplementary material for: Early detection of clinically significant prostate cancer: protocol summary and statistical analysis plan for the ProScreen randomised trial
Source: BMJ Open. 2024 Jan 9;14(1):e075595. doi: 10.1136/bmjopen-2023-075595 (PMC10806703; doi:10.1136/bmjopen-2023-075595)
Supplement: Author's manuscript [file bmjopen-2023-075595.draft_revisions.pdf]

BMJ Open

BMJ Open is committed to open peer review. As part of this commitment we make the peer review history of every article we publish publicly available.

When an article is published we post the peer reviewers' comments and the authors' responses online. We also post the versions of the paper that were used during peer review. These are the versions that the peer review comments apply to.

The versions of the paper that follow are the versions that were submitted during the peer review process. They are not the versions of record or the final published versions. They should not be cited or distributed as the published version of this manuscript.

BMJ Open is an open access journal and the full, final, typeset and author-corrected version of record of the manuscript is available on our site with no access controls, subscription charges or pay-per-view fees (http://bmjopen.bmj.com).

If you have any questions on BMJ Open's open peer review process please email info.bmjopen@bmj.com

BMJ Open

Protocol summary and statistical analysis plan for the randomized trial of early detection of clinically significant prostate cancer (ProScreen)

Journal:	*BMJ Open*
Manuscript ID	bmjopen-2023-075595
Article Type:	Protocol
Date Submitted by the Author:	12-May-2023
Complete List of Authors:	Nevalainen, Jaakko; Tampere University<br>Raitanen, Jani; Tampere University, Faculty of Social Sciences (Health Sciences) and Gerontology Research Center<br>Natunen, Kari; Tampere University<br>Kilpelainen, Tuomas; University of Helsinki; Helsinki University Central Hospital<br>Rannikko, Antti; University of Helsinki; Helsinki University Central Hospital<br>Tammela, Teuvo; Tampere University; Tampere University Hospital<br>Auvinen, Anssi; Tampere University
Keywords:	Mass Screening, Prostatic Neoplasms, Randomized Controlled Trial, STATISTICS & RESEARCH METHODS

SCHOLARONE™
Manuscripts

Title: Protocol summary and statistical analysis plan for the randomized trial of early detection of clinically significant prostate cancer (ProScreen)

Authors: Jaakko Nevalainen[1], Jani Raitanen[1], Kari Natunen[1], Tuomas Kilpeläinen[2,3], Antti Rannikko[2,3], Teuvo Tammela[4], Anssi Auvinen[1], and the ProScreen Trial Team

Affiliations

1. Unit of Health Sciences, Faculty of Social Sciences, Tampere University, Tampere, Finland
2. Department of Urology, University of Helsinki and Helsinki University Hospital, Helsinki, Finland
3. Research Program in Systems Oncology, Faculty of Medicine, University of Helsinki, Helsinki, Finland
4. Tampere University Hospital, Department of Urology and Tampere University, Faculty of Medicine and Health Technology, Tampere, Finland

For peer review only

ABSTRACT

Introduction: Evidence on the effectiveness of prostate cancer screening based on prostate-specific antigen is inconclusive and suggests a questionable balance between benefits and harms due to overdiagnosis. However, diagnostic accuracy studies have shown that detection of clinically insignificant prostate cancer can be reduced by magnetic resonance imaging combined with targeted biopsies.

The aim of the paper is to describe the analysis of the ProScreen randomized trial to assess the performance of the novel screening algorithm in terms of the primary outcome, prostate cancer mortality, and secondary outcomes as intermediate indicators of screening benefits and harms of screening.

Methods: The trial aims to recruit at least 111,000 men to achieve sufficient statistical power for the primary outcome. Men will be allocated in a 1:3 ratio to the screening and control arms. Interim analysis is planned at 10 years of follow-up, and the final analysis at 15 years. Difference between the trial arms in prostate cancer mortality will be assessed by Gray's test using intention to screen analysis of randomized men. Secondary outcomes will be the incidence of prostate cancer by disease aggressiveness, progression to advanced prostate cancer, death due to any cause and cost-effectiveness of screening.

Ethics and dissemination: The trial protocol was reviewed by the ethical committee of the Helsinki University Hospital (HUS 2910/2017). Results will be disseminated in an international peer-reviewed journal(s) and at scientific meetings.

Trial Registration: NCT03423303

Keywords: effectiveness; prostate cancer screening; randomized trial; screening algorithm

STRENGTHS AND LIMITATIONS

- This population-based, randomized multicenter trial targeting at recruiting 111,000 men will provide high quality evidence on the effectiveness of a novel screening strategy for prostate cancer mortality
- Broad eligibility criteria and pragmatic approach embedded in normal clinical practice enhances the external validity of the trial and provide evidence applicable to decision making in public health and health care
- Challenges for the trial include the maintenance of high compliance to screening and the extent of opportunistic PSA testing in the population

For peer review only

Introduction

Prostate cancer is the most common cancer in men in many industrialized countries and causes substantial mortality (Culp et al. 2020). Screening based on blood prostate-specific antigen (PSA) has been shown to decrease prostate cancer mortality, but the evidence from randomized trials is not conclusive (Hugosson et al. 2019, Pinsky et al. 2019). Systematic reviews of randomized controlled trials have concluded that PSA screening may at best lower prostate cancer mortality, but not all-cause mortality. However, the balance between benefits and harms was regarded as problematic due to frequent overdiagnosis, and complications from biopsies and overtreatment (Ilic et al. 2018, Fenton et al. 2018, Paschen et al. 2022).

Several studies have shown that detection of clinically insignificant prostate cancer can be reduced by magnetic resonance imaging (MRI) combined with targeted biopsies of the suspect foci, instead of systematic biopsies of the entire prostate (Schoots et al. 2015; Ahmed et al. 2017). However, previous studies have mostly focused on the diagnostic performance, i.e., cancer detection at a single evaluation. A hybrid screening/diagnostic study and a screening trial using MRI were recently published (Hugosson et al. 2022, Eklund et al. 2021).

Here we describe the analysis of the ProScreen randomized screening trial to assess the performance of a novel screening algorithm in terms of the primary outcome, prostate cancer mortality, and secondary outcomes, used as intermediate indicators of benefits and harms of screening. Following good statistical practice, this statistical analysis plan (version 1.0) was finalized prior to completion of recruitment and short-term follow-up data collection. It was written following the guidelines provided in Gamble *et al.* (2017) as applicable. Any unforeseen deviations from the plan will be described and justified carefully in the respective reports.

Trial overview

Trial design

The ProScreen trial is a population-based, randomized multicenter trial that investigates the effectiveness of a novel screening strategy combining PSA, a four-kallikrein panel, and MRI on prostate cancer (PCa) mortality over a 15-year period from randomization (Auvinen et al. 2017). The rationale is to minimize detection of clinically insignificant cancers, while maintaining a high sensitivity for aggressive cancers in order to reduce overdiagnosis without compromising mortality benefits. An interim analysis of PCa mortality is planned at 10 years of follow-up.

On 15 January 2018, the trial was registered at clinicaltrials.gov (NCT03423303). The ethical committee of Helsinki University Hospital reviewed the protocol (tracking no. 2910/2017). Permissions to collect data from health care registers was obtained from Finnish Institute for Health and Welfare (before the era of FinData, Dnro THL/676/5.05.00/2018). A written informed consent is provided by each participant in the screening arm.

Recruitment started in October 2018 and is still ongoing.

Study population

All men aged 50–63 years (at the time of sampling of the trial population) with Finnish or Swedish as mother tongue residing in the trial municipalities constitute the trial population. Men with a prevalent prostate cancer will be identified through the Finnish Cancer Registry or hospital pathology databases and excluded.

For peer review only

We have identified for the trial the entire target population from the Digital and Population Data Services Agency, comprehensively without any sampling. The initial trial population consists of men residing in Helsinki and Tampere.

Currently, we are increasing the sample size by recruiting men also in the other municipalities within the Helsinki and Tampere metropolitan areas (Vantaa, Espoo, and Kauniainen, as well as Nokia, Lempäälä, Pirkkala, Ylöjärvi, and Kangasala with a total of 57,000 men in the target age group). The target population covers comprehensively all eligible men in the municipalities, both in the original Helsinki and Tampere areas and the new municipalities.

Sample size

We estimated that we could find 110,000–120,000 men in the target age group based on the population projections from 2020 to 2034 from the ten municipalities (Statistics Finland 2021). We requested the overall number of deaths and the number of PCa deaths from Statistics Finland by age group from 1990 to 2019. The proportion of PCa deaths had barely changed at all during the 30-year period and hence, we based our sample size calculation on these figures. With a 1:3 random allocation to the screening arm relative to the control arm, we estimated that at least 240 PCa deaths would occur in the control arm during the first ten years of the trial, and at least 520 PCa deaths by 15 years of follow-up.

Assuming a relative hazard of 0.75 for the screening relative to the control arm, Schoenfeld's formula indicates that an 80% power would be reached by a total of 506 PCa deaths (Schoenfeld 1983) with type I error rate set at 5%. Assuming a total of 650 PCa deaths – 520 in the control arm and 130 in the screening arm – the power of the study would be 89%. Hence, we aim at a final sample size of at least 111,000 men to ensure adequate statistical power and precision at the final analysis.

Randomization and screening intervals

All eligible men will be randomly allocated to screening and control arms in a 1:3 ratio. Within the screening arm, re-screening interval is adapted by the baseline PSA:

- Men with initial PSA≥3 ng/ml are re-invited every two years,
- Men with PSA 1.5–2.99 ng/ml every four years, and
- Men with PSA<1.5 ng/ml after six years.

By the time of writing this plan, we have randomized 61,193 men with 15,299 allocated to the screening arm and 45,894 to the control arm. Analyses will compare the entire screening arm, regardless of the actual screening attendance and interval employed, to the control arm, unless otherwise specified.

Randomization list consists of batches of randomized men. The list is generated centrally by a designated study biostatistician at the coordinating unit, who maintains the documentation including program codes and the resulting lists include information of randomization dates, personal identification numbers (linkable to study ID number) and the arm allocated. Randomization lists are only shared confidentially to study personnel if needed for study conduct.

For peer review only

Screening procedures

At every screening attendance, three consecutive tests are conducted in a stepwise manner before biopsy:

1. All participating men give a blood sample for determination of PSA at a local laboratory.
2. If the PSA is 3 ng/ml or higher, a four-kallikrein panel is analyzed from a second vial of plasma from the initial draw using an algorithm incorporating four proteins (total PSA, free PSA, intact PSA and human kallikrein-2) and age. The result is expressed as probability of a clinically significant PCa.
3. Men with both PSA≥3 and kallikrein score≥7.5% are referred to MRI. T2-weighted, diffusion-weighted and dynamic contrast-enhanced imaging is employed in accordance with the European Society for Urogenital Radiology guideline (de Rooij 2020). The findings are classified according to the Prostate Imaging Reporting and Data System (PI-RADS v2.1), which is a 5-point scale to combine the MRI findings and indicate the likelihood of a significant cancer. Scores of 3–5 indicate at least a suspect finding warranting directed biopsy.

Only targeted biopsies are employed, with 2–4 cores per region of interest depending on the size. Only screen-positive men with negative MRI but PSA density >0.15 undergo systematic biopsy as a safety measure (to avoid missing clinically significant cancers). Similar fusion-guided biopsy systems are used at the two trial sites and evaluated by experienced uropathologists using standardized procedures.

A random sample of screen-negative (on test steps 1 and 2) men are also invited to prostate MRI and asked to give blood, urine and stool samples in order to serve as a control group to estimate frequency of suspicious MRI findings in the general population, and as a reference group in analyses of biological samples.

Protocol deviations

A tabular presentation of different types of protocol deviations along with their frequencies and percentages will be presented. Any protocol deviations detected after randomization will be carefully documented. Among them, men later found out not to have met the eligibility criteria at the date of randomization can be excluded from the analysis (post-randomization exclusions).

In the case of major protocol violations affecting a substantial proportion of men, separate per protocol analyses will be conducted to support the main analyses. In the screening arm, incomplete attendance, or compliance with the screening procedures is likely to occur. In the control arm, we will obtain data on contamination, i.e., mostly self-initiated PSA testing.

When considering unforeseen lack of compliance with the protocol, all means to ensure objectivity in the exclusion principles from per protocol analyses will be taken. Participants in both arms will be considered according to the same principles. Protocol deviations not related to the screening procedures are expected to appear in approximately 1:3 ratio for the arms. Obvious deviation from this ratio would be reported and interpreted as a potential source of bias.

For peer review only

Blinding

Blinding in the conventional sense is not applicable: men are aware of being invited to screening. Hence, this is an open trial with screening and control arms.

Concrete measures to prevent bias, if any, from the awareness of the trial arm were nevertheless taken: (i) the control arm is blind to the fact that they are part of the trial; (ii) allocation concealment is ensured by the centralized randomization procedure preventing foreknowledge of upcoming arm allocation; and (iii) communication to the general public on trial is kept to the minimum to prevent contamination (e.g. by self-initiated PSA testing) among men in the control arm.

In addition, we underline that the primary outcome of the study, PCa death, is an objective outcome. The possibility of bias in its evaluation only relates to the assessment of the cause of death. The death certificates are filled by physicians with no involvement in the trial and can be assumed to be independent of trial arm, especially as deaths from prostate cancer are likely to occur years after the diagnosis and hence unaffected by detection through screening or other means. Importantly, a previous study within the ERSPC trial has shown that the cause-of-death data provided by Statistics Finland agreed almost perfectly with the assessment of a blinded expert panel in the Finnish center of the trial and was independent of the trial arm (Mäkinen et al. 2008, Kilpeläinen et al. 2016).

Data collection process

Table 1 summarizes the stages of the data collection process, targeted participants, and information and samples obtained.

Table 1. Data collection process of the ProScreen trial.

Process stage	Target population	Information collected	Samples collected
**Baseline**	Participants	Family history<br>Previous PSA and Bx<br>Generic QoL/utility (15D, EQ5D)<br>Out-of-pocket costs<br>PSA and four-kallikrein panel	Plasma<br>Serum<br>Whole blood
**MRI**	Men with PSA >3 ng/ml and kallikrein score >7.5%	PIRADS score	Digital image
**Biopsy**	Screen-positive men	Post-biopsy symptoms (0, 30 days)<br><br>Targeted fusion biopsies: number of ROIs, number of biopsies, length of samples,<br><br>Systemic biopsies: Biopsy length, cancer length and Gleason score per sample, total length of samples, total length of cancer, portion of cancer, global Gleason score, portion of Gleason 4 or 5, perineural invasion	Urine<br>Stool<br>RNA, DNA<br>Cancer tissue and prostate tissue<br>Plasma<br>Serum<br>Whole blood

For peer review only - http://bmjopen.bmj.com/site/about/guidelines.xhtml

**Cancer diagnosis**	Men with prostate cancer	Disease-specific QoL (EPIC-26, MAX-PC)<br><br>Generic Qol/utility (EQ5D, 15D), out-of-pocket costs<br><br>Gleason/ISUP grade group, number of positive cores, length of cancer, treatment, TNM stage	

Study outcomes and other relevant variables

The primary outcome of the trial is death from prostate cancer. Causes of death will be obtained from the Statistics Finland database and the underlying causes of death will be considered when evaluating if the man died from PCa or from other causes. Cancer cases in the entire trial population including the control arm and non-participants in the screening arm are identified from pathology databases of the two hospitals and through linkages to the Finnish Cancer Registry using the unique PID assigned to all Finnish residents to ensure complete coverage and avoid duplicates (double count).

Secondary outcomes are:

- Diagnosis of prostate cancer (divided into clinically significant and insignificant)
- Progression to advanced prostate cancer (biochemical relapse or progression to metastatic)
- Death due to any cause
- Cost-effectiveness of screening

Adverse outcome variables to monitor screening-related harms are:

- Overdiagnosis of clinically insignificant prostate cancer
- Quality of life impacts of screening and quality of life among men with PCa (EPIC26 instrument)
- Prostate cancer-related anxiety (MAX-PC questionnaire)
- Complications from biopsy (PRECISION questionnaire)

Statistical analysis

The main analyses will rely on the intention to screen (ITS) principle and will include all randomized men in the two trial arms who were alive and eligible (free of prostate cancer) at the date of randomization. Those men who became ineligible between the date of randomization and first screening invitation will remain in the ITS analysis set.

Two-sided statistical tests will be used, and the overall significance level will be set at 5%. Corresponding p-values will be accompanied with estimates of differences and their 95% confidence intervals.

Analysis of the primary outcome

The primary outcome of the trial is death from prostate cancer. This is a superiority trial regarding the primary outcome and the comparisons between trial arms will be analyzed and presented on this basis.

For peer review only

Those men who survived will be considered as right-censored observations at the time passed between the time of analysis and time of randomization period. Those men who were lost to follow-up (e.g., due to emigration) will be considered as censored at that particular time (e.g., at emigration). Time to death, defined as the difference between the date of death and date of randomization, will be used as the event time for the analysis.

To evaluate differences between screening and control arms in prostate cancer specific mortality, Gray's test (Gray 1988) for testing the null hypothesis of equality of cumulative incidence functions will be used. This test differs from the commonly used logrank test in how competing risks of death are treated and is based on the subdistribution hazard of prostate cancer cause of death.

The test will be complemented by reporting the number of PCa deaths, number of men at risk and estimated cumulative incidence functions for each trial arm over follow-up time. The arms will be compared in absolute risks (number needed to invite i.e., the inverse of the risk difference and number needed to diagnose per averted prostate cancer death, i.e., the ratio of excess incidence to mortality reduction), as well as and relative measures of effect (hazard ratios). Descriptive summaries will also be presented by trial centers, age group at randomization.

<u>Secondary analyses of the primary outcome</u>

Fine-Gray model for the subdistribution hazard will be used to conduct analyses adjusted for background factors. Outcomes will be compared between age groups and trial centers, and in case of differences, analyses to control for trial center and for age at randomization (categorized as 50–54, 55–59, 60–65 years) will be conducted.

Per protocol analyses excluding men with substantial protocol deviations, such as repeated non-attendance or ineligibility before invitation to screening (with pseudo invitation dates for the control arm), will be conducted if considered pertinent. Additional analyses to correct for contamination and non-compliance, i.e., estimation of efficacy, will be taken by best practices methods at the time of the analyses (e.g., Cuzick et al. 1997).

Descriptive analyses to study effect heterogeneity by center and age group will be performed to complement these analyses. Additional analyses requested by external reviewers or editors in peer-review processes will also be done.

Analysis of secondary outcomes

<u>Diagnosis of prostate cancer</u>

The analysis of cumulative incidence of PCa by disease aggressiveness intends to assess screening impact on detection of clinically significant PCa (representing potential benefit through early treatment) and clinically insignificant PCa (indicating overdiagnosis). The intention is to assess the extent of detection of clinically significant PCa by screening relative to the control arm, and extent of overdiagnosis relative to the control arm. This will inform about the degree of accomplishing rationale of the trial, i.e., detection of aggressive cases at least similar to that in PSA-based screening, while substantially decreasing the yield of low-risk cases. As screening advances the time of diagnosis by several years (lead time), cumulative incidence will be used as the indicator of risk.

Disease aggressiveness will be defined by the International Society for Urological Pathology (ISUP) Gleason grade group. The analyses will be conducted separately for the detection of clinically significant (Gleason 7+ or ISUP 2+) and clinically insignificant (Gleason <7 or ISUP

For peer review only

1) PCa. In secondary analyses, alternative criteria for csPCa will also be employed including ISUP 3+ (Gleason 4+3 or higher), maximum length of cancer tissue in biopsy and number of biopsy cores with cancer.

Risk differences and ratios will be used to infer screening benefits and overdiagnosis compared to the control arm. Besides cumulative incidence, the ratio of aggressive to non-aggressive cases (or proportion of aggressive cancers out of all PCa) will also be reported.

Cumulative incidence for both outcomes will be estimated by trial arm. The overall PCa incidence combines screening benefits and harms and is thus regarded of minor importance in the interpretation of screening impact. Tabular presentations of age at diagnosis, disease stage and grade at diagnosis will be presented.

Both intention to screen (by allocation) and per protocol (screening participants and non-participants) analyses will be conducted for each screening round. For screening participants, screen-detected and interval cases will be reported separately, and screen-detected cases will be broken down by those detected in targeted biopsies of MRI-positive lesions (screening protocol evaluated) and systematic biopsies in screen-negative men with PSA density >0.15 (safety measure to avoid missing clinically significant cases). Any cases detected in a random sample of screen-negative men invited to MRI (analyses to assess underlying prevalence of prostate cancer) will also be reported separately. Analyses to evaluate an optimized screening algorithm will include exclusion of cases with PI-RADS score 3 and kallikrein score calculated also incorporating information on previous biopsies (ignored in the main analysis), as well as using higher cut-off values for PSA and the kallikrein score.

Advanced prostate cancer

The analysis of advanced prostate cancer will compare the cumulative incidence of cancer progression, including metastasis and/or biochemical relapse developing after diagnosis and primary treatment, between the screening and control arms. The purpose of the analysis is to evaluate differences between the arms in the risk of developing a potentially lethal, advanced PCa.

The origin of the analysis will be the time of randomization. Cumulative incidence rates will be estimated by the Kaplan-Meier method, and differences between trial arms will be estimated by Cox regression models adjusted by age at diagnosis.

Death due to any cause

The analysis of all-cause mortality aims to show that the trials arms are comparable with each other and the general male population in Finland. These analyses will not inform about the effectiveness of screening. Cumulative survival and mortality rates will be estimated by the Kaplan-Meier method, from time of randomization, displayed with frequencies of events and men at risk by trial arm, and by age at randomization.

This analysis will focus on the intention to screen analysis set.

Cost-effectiveness

A cost-effectiveness analysis will be performed, incorporating cost data for both out-of-pocket estimated from surveys and service cost data collected from health care providers, as well mortality results (ITS analysis) and utilities based on repeated surveys with 15D and EQ5D instruments (on a random sample of participants). The comparator is no active screening, here represented by the control arm. The main outcome is the incremental cost-effectiveness ratio in terms of costs per quality-adjusted life-year.

For peer review only - http://bmjopen.bmj.com/site/about/guidelines.xhtml

A preliminary and exploratory cost effectiveness study can be conducted after the last of the follow-up surveys have been returned, approximately at 3 years after the randomization of the last man into the trial. We plan to undertake a full cost-effectiveness analysis around the time when the evidence on the effectiveness of screening regarding primary outcome has been obtained; this will most likely be near to the analysis at 15 years.

Quality of life

These analyses aim to evaluate the short-term and long-term impacts of screening on generic quality of life as well as disease-specific quality of life among men with PCa. Two disease-specific questionnaires, EPIC26 instrument and MAX-PC questionnaires will be used to measure quality of life at 0, 6, 12, and 24 months from PCa diagnosis in both trial arms.

Standard scoring of the EPIC26 instrument will be used. Summary statistics of the five key domains over time and by trial arm will be calculated to assess changes in quality of life of men with PCa from diagnosis onwards. Summary and domain-grouped scores will be analyzed using applications of linear models (or their nonparametric counterparts, if needed) for repeated measures to evaluate differences in quality of life between the arms following PCa diagnosis.

Prostate cancer related anxiety is measured with the Memorial Anxiety Scale for Prostate Cancer (MAX-PC) questionnaire (Roth et al. 2003). Results will be presented as frequencies and percentages for total and subscale scores by trial arm.

Generic quality of life and utilities are evaluated using the 15D and EQ5D instruments as described in the cost-effectiveness section.

Analysis of adverse outcomes

In addition to detection of low-risk disease by screening as an indicator of overdiagnosis, adverse outcomes mainly relate to the harms due to biopsies. Adverse effects of prostate biopsy are monitored using the questionnaire developed for the PRECISION trial covering pain and other symptoms immediately after biopsy and at 30 days following biopsy. The number of biopsies, as well as the number (%) and type of complications among those with biopsies will be reported.

Interim analyses and data monitoring

The first analysis of PCa mortality will be conducted at 10 years and the final analysis at 15 years (i.e. at median follow-up time of 10 or 15 years). As we do not intend to stop the trial at 10 years, these interim analyses will be considered as preliminary information. Interim analyses at 10 years will include also analyses of shorter-term benefits.

To control the overall type I error rate (5%) of the trial, we will employ the O'Brien-Fleming rule for alpha spending function. We set the amount of information at 0.5 at 10 years based on the expected numbers of PCa deaths. Thus, by implementation of the O'Brien-Fleming algorithm, the resulting significance level at 10-year interim analysis will be 0.0056, and at the 15-year final analysis 0.0444.

Analyses of secondary endpoints informing about the intermediate outcomes of process indicators including participation, cancer detection, validity and diagnostic performance of the tests in the entire population and subgroups (screened men, non-participants, men in the control arm) will be carried out at regular intervals, as sufficient data become available for evaluation. These will inform about potential need to modify the procedures. Side studies using the samples collected will be carried out to identify new indicators of prostate cancer risk and prognosis.

For peer review only

An independent data monitoring committee (DMC) oversees the trial conduct, and its main task is to ensure safety of the participants. Safety in this context means that screening or screening procedures should not lead to unacceptable disadvantage for the participants in the light of screening benefits. This could take place if the screening intervention had materially worse performance in detecting clinically relevant prostate cancer than anticipated, or substantially higher level of overdiagnosis. The DMC is given a report of the screening results initially every six months and after the first year every 12 months. The DMC can also request any additional information they regard as pertinent to their task. In case of concern, the DMC can recommend discontinuation of the trial; in practice that would mean stopping recruitment and discontinuation of further screening procedures. In addition, they have a mandate to suggest modifications to the trial protocol.

Handling of missing data

Extent of missing data will be described, for example, by presenting the number of individuals with missing values per variable.

For outcome variables relying on dates – dates of randomization, censoring, diagnosis or death – incomplete dates will be imputed by 15 (in the case that the day variable was missing, but known month and year), and by 30/6 (in the case that only the year was known).

In case a substantial proportion of men (at least 5%) have missing data on one or more variable needed for the effectiveness analysis in question, multiple imputation methods will be used to demonstrate the robustness of findings (Little *et al.* 2012). Imputation models will include outcome variables and trial arm in addition to all variables relevant to the particular analysis. Final estimates will be derived by combining estimates and their standard errors across data sets using Rubin's rules.

Data management and quality assurance

RedCap database application is used for data management in the trial, covering all major data types from questionnaires and lab results to MRI findings, diagnoses and causes of death. RedCap allows access defined by two-factor authentication (2FA) and flexible definition of user-specific functions and rights.

In REDCap, variable specific parameters and predetermined options are used to prevent entering invalid data (e.g. predefined values and acceptable ranges). All data is verified from the original data source and monitored monthly. Until the verification, data is saved as incomplete or unverified. Lead times between screening tests are monitored every 6-8 weeks. For the laboratory work (including sampling, processing, and storing) each task has a protocol shared by the study centers. Any deviations from the sample specific protocol are documented.

Conclusion

This statistical analysis plan lays out the plans for outcomes of the trial, including the definitions of important outcomes, analysis principles and interpretation, methods for primary analysis, pre-specified subgroup analysis, and secondary analysis.

References

Ahmed HU, El-Shater Bosaily A, Brown LC, *et al*. Diagnostic accuracy of multi-parametric MRI and TRUS biopsy in prostate cancer (PROMIS): a paired validating confirmatory study. *Lancet* 2017; 389:815-822. doi: 10.1016/S0140-6736(16)32401-1. PMID: 28110982.

For peer review only

Auvinen A, Rannikko A, Taari K, *et al.* A randomized trial of early detection of clinically significant prostate cancer (ProScreen): study design and rationale. *Eur J Epidemiol* 2017; 32:521-527. doi: 10.1007/s10654-017-0292-5. PMID: 28762124.

Culp MB, Soerjomataram I, Efstathiou JA, *et al.* Recent global patterns in prostate cancer incidence and mortality rates. *Eur Urol* 2020; 77:38-52

Cuzick J, Edwards R, Segnan N. Adjusting for non-compliance and contamination in randomized clinical trials. *Stat Med* 1997; 16:1017-29. doi: 10.1002/(sici)1097-0258(19970515)16:9<1017::aid-sim508>3.0.co;2-v. Erratum in: *Stat Med* 2007;26:3821. PMID: 9160496.

Eklund M, Jäderling F, Discacciati A, *et al*. MRI-Targeted or Standard Biopsy in Prostate Cancer Screening. *N Engl J Med* 2021; 385:908-920. doi: 10.1056/NEJMoa2100852. PMID: 34237810.

Fenton JJ, Weyrich MS, Durbin S, *et al*. Prostate-specific antigen-based screening for prostate cancer. *JAMA* 2018; 319:1914-31.

Gamble C, Krishan A, Stocken D, *et al*.. Guidelines for the content of statistical analysis plans in clinical trials. *JAMA* 2017; 318:2337–43. https://doi.org/10.1001/jama.2017.18556.

Gray RJ: A class of K-sample tests for comparing the cumulative incidence of a competing risk. *Ann Stat* 1988; 16:1141-1154.

Hugosson J, Roobol MJ, Månsson M, *et al*. 16-year follow-up of the European Randomised Study of Screening for Prostate Cancer (ERSPC). *Eur Urol* 2019; 76:43-51.

Hugosson J, Månsson M, Wallström J, *et al.* Prostate Cancer Screening with PSA and MRI Followed by Targeted Biopsy Only. *N Engl J Med*. 2022; 387:2126-2137. doi: 10.1056/NEJMoa2209454. PMID: 36477032.

Ilic D, Djulbegovic M, Jung JH, *et al.* Prostate cancer screening with prostate-specific antigen (PSA) test: a systematic review and meta-analysis. *BMJ* 2018; 362:k3519. doi: 10.1136/bmj.k3519. PMID: 30185521.

Kilpeläinen TP, Mäkinen T, Karhunen PJ, *et al.* Estimating bias in causes of death ascertainment in the Finnish Randomized Study of Screening for Prostate Cancer. *Cancer Epidemiol* 2016; 45:1-5. doi: 10.1016/j.canep.2016.08.022. PMID: 27636505.

Little RJ, D'Agostino R, Cohen ML, *et al.* The prevention and treatment of missing data in clinical trials. *N Engl J Med* 2012; 367:1355-60. doi: 10.1056/NEJMsr1203730. PMID: 23034025.

Mäkinen T, Karhunen P, Aro J, *et al.* Assessment of causes of death in a prostate cancer screening trial. Int J Cancer 2008; 122: 413-417. https://doi.org/10.1002/ijc.23126

Paschen U, Sturz S, Fleer D, *et al*. Assessment of prostate-specific antigen screening. *BJU Int* 2022;129:280-289

For peer review only

For peer review only - http://bmjopen.bmj.com/site/about/guidelines.xhtml

Pinsky PF, Miller E, Prorok P, *et al*. Extended follow-up for prostate cancer incidence and mortality among participants in the PLCO screening trial. *BJU Int* 2019;123:854-860

Schoots IG, Roobol MJ, Nieboer D, *et al*. Magnetic resonance imaging-targeted biopsy may enhance the diagnostic accuracy of significant prostate cancer detection compared to standard transrectal ultrasound-guided biopsy: a systematic review and meta-analysis. *Eur Urol* 2015; 68:438-50. doi: 10.1016/j.eururo.2014.11.037. PMID: 25480312.

Rannikko A, Leht M, Mirtti T, *et al*. Population-based randomized trial of screening for clinically significant prostate cancer ProScreen: a pilot study. *BJU Int* 2022; 130:193-199. doi: 10.1111/bju.15683. PMID: 34958531.

de Rooij M, Israël B, Tummers M, *et al.* ESUR/ESUI consensus statements on multi-parametric MRI for the detection of clinically significant prostate cancer: quality requirements for image acquisition, interpretation and radiologists' training. *Eur Radiol* 2020; 30:5404-5416. doi: 10.1007/s00330-020-06929-z. PMID: 32424596.

Roth AJ, Rosenfeld, B, Kornblith AB, *et al.* The memorial anxiety scale for prostate cancer. *Cancer* 2003; 97:2910-18

Statistics Finland's StatFin online service. Available at: https://statfin.stat.fi/PXWeb/pxweb/en/StatFin/ (Accessed 9 June 2021).

Schoenfeld DA. Sample-size formula for the proportional-hazards regression model. *Biometrics* 1983; 39:499-503.

Authors' contributions

All authors approved the final version of this manuscript. Study concept and design: AA, AR, JN, JR, KN, TK, TT; drafting of the manuscript: AA, JN; critical revision of the manuscript for important intellectual content: AA, AR, JN, JR, KN, TK, TT.

Funding statement

This work was supported by the Academy of Finland (grant number 311336) the Finnish Cancer Foundation, the Jane and Aatos Erkko foundation, Competitive State Research Funding administered by Tampere University Hospital (grant number 9V02), and Päivikki and Sakari Sohlberg Foundation.

For peer review only

Title: Protocol summary and statistical analysis plan for the randomized trial of early detection of clinically significant prostate cancer (ProScreen)

Authors: Jaakko Nevalainen[1], Jani Raitanen[1], Kari Natunen[1], Tuomas Kilpeläinen[2,3], Antti Rannikko[2,3], Teuvo Tammela[4], Anssi Auvinen[1], and the ProScreen Trial Team

Affiliations

1. Unit of Health Sciences, Faculty of Social Sciences, Tampere University, Tampere, Finland
2. Department of Urology, University of Helsinki and Helsinki University Hospital, Helsinki, Finland
3. Research Program in Systems Oncology, Faculty of Medicine, University of Helsinki, Helsinki, Finland
4. Tampere University Hospital, Department of Urology and Tampere University, Faculty of Medicine and Health Technology, Tampere, Finland

For peer review only

ABSTRACT

Introduction: Evidence on the effectiveness of prostate cancer screening based on prostate-specific antigen is inconclusive and suggests a questionable balance between benefits and harms due to overdiagnosis. However, diagnostic accuracy studies have shown that detection of clinically insignificant prostate cancer can be reduced by magnetic resonance imaging combined with targeted biopsies.

The aim of the paper is to describe the analysis of the ProScreen randomized trial to assess the performance of the novel screening algorithm in terms of the primary outcome, prostate cancer mortality, and secondary outcomes as intermediate indicators of screening benefits and harms of screening.

Methods: The trial aims to recruit at least 111,000 men to achieve sufficient statistical power for the primary outcome. Men will be allocated in a 1:3 ratio to the screening and control arms. Interim analysis is planned at 10 years of follow-up, and the final analysis at 15 years. Difference between the trial arms in prostate cancer mortality will be assessed by Gray's test using intention to screen analysis of randomized men. Secondary outcomes will be the incidence of prostate cancer by disease aggressiveness, progression to advanced prostate cancer, death due to any cause and cost-effectiveness of screening.

Ethics and dissemination: The trial protocol was reviewed by the ethical committee of the Helsinki University Hospital (HUS 2910/2017). Results will be disseminated in an international peer-reviewed journal(s) and at scientific meetings.

Trial Registration: NCT03423303

Keywords: effectiveness; prostate cancer screening; randomized trial; screening algorithm

STRENGTHS AND LIMITATIONS

- This population-based, randomized multicenter trial targeting at recruiting 111,000 men will provide high quality evidence on the effectiveness of a novel screening strategy for prostate cancer mortality
- Broad eligibility criteria and pragmatic approach embedded in normal clinical practice enhances the external validity of the trial and provide evidence applicable to decision making in public health and health care
- Challenges for the trial include the maintenance of high compliance to screening and the extent of opportunistic PSA testing in the population

For peer review only

Introduction

Prostate cancer is the most common cancer in men in many industrialized countries and causes substantial mortality (Culp et al. 2020). Screening based on blood prostate-specific antigen (PSA) has been shown to decrease prostate cancer mortality, but the evidence from randomized trials is not conclusive (Hugosson et al. 2019, Pinsky et al. 2019). Systematic reviews of randomized controlled trials have concluded that PSA screening may at best lower prostate cancer mortality, but not all-cause mortality. However, the balance between benefits and harms was regarded as problematic due to frequent overdiagnosis, and complications from biopsies and overtreatment (Ilic et al. 2018, Fenton et al. 2018, Paschen et al. 2022).

Several studies have shown that detection of clinically insignificant prostate cancer can be reduced by magnetic resonance imaging (MRI) combined with targeted biopsies of the suspect foci, instead of systematic biopsies of the entire prostate (Schoots et al. 2015; Ahmed et al. 2017). However, previous studies have mostly focused on the diagnostic performance, i.e., cancer detection at a single evaluation. A hybrid screening/diagnostic study and a screening trial using MRI were recently published (Hugosson et al. 2022, Eklund et al. 2021).

Here we describe the analysis of the ProScreen randomized screening trial to assess the performance of a novel screening algorithm in terms of the primary outcome, prostate cancer mortality, and secondary outcomes, used as intermediate indicators of benefits and harms of screening. Following good statistical practice, this statistical analysis plan (version 1.0) was finalized prior to completion of recruitment and short-term follow-up data collection. It was written following the guidelines provided in Gamble *et al.* (2017) as applicable. Any unforeseen deviations from the plan will be described and justified carefully in the respective reports.

Trial overview

Trial design

The ProScreen trial is a population-based, randomized multicenter trial that investigates the effectiveness of a novel screening strategy combining PSA, a four-kallikrein panel, and MRI on prostate cancer (PCa) mortality over a 15-year period from randomization (Auvinen et al. 2017). The rationale is to minimize detection of clinically insignificant cancers, while maintaining a high sensitivity for aggressive cancers in order to reduce overdiagnosis without compromising mortality benefits. An interim analysis of PCa mortality is planned at 10 years of follow-up.

On 15 January 2018, the trial was registered at clinicaltrials.gov (NCT03423303). The ethical committee of Helsinki University Hospital reviewed the protocol (tracking no. 2910/2017). Permissions to collect data from health care registers was obtained from Finnish Institute for Health and Welfare (before the era of FinData, Dnro THL/676/5.05.00/2018). A written informed consent is provided by each participant in the screening arm.

Recruitment started in October 2018 and is still ongoing.

Study population

All men aged 50–63 years (at the time of sampling of the trial population) with Finnish or Swedish as mother tongue residing in the trial municipalities constitute the trial population. Men with a prevalent prostate cancer will be identified through the Finnish Cancer Registry or hospital pathology databases and excluded.

For peer review only

We have identified for the trial the entire target population from the Digital and Population Data Services Agency, comprehensively without any sampling. The initial trial population consists of men residing in Helsinki and Tampere.

Currently, we are increasing the sample size by recruiting men also in the other municipalities within the Helsinki and Tampere metropolitan areas (Vantaa, Espoo, and Kauniainen, as well as Nokia, Lempäälä, Pirkkala, Ylöjärvi, and Kangasala with a total of 57,000 men in the target age group). The target population covers comprehensively all eligible men in the municipalities, both in the original Helsinki and Tampere areas and the new municipalities.

Sample size

We estimated that we could find 110,000–120,000 men in the target age group based on the population projections from 2020 to 2034 from the ten municipalities (Statistics Finland 2021). We requested the overall number of deaths and the number of PCa deaths from Statistics Finland by age group from 1990 to 2019. The proportion of PCa deaths had barely changed at all during the 30-year period and hence, we based our sample size calculation on these figures. With a 1:3 random allocation to the screening arm relative to the control arm, we estimated that at least 240 PCa deaths would occur in the control arm during the first ten years of the trial, and at least 520 PCa deaths by 15 years of follow-up.

Assuming a relative hazard of 0.75 for the screening relative to the control arm, Schoenfeld's formula indicates that an 80% power would be reached by a total of 506 PCa deaths (Schoenfeld 1983) with type I error rate set at 5%. Assuming a total of 650 PCa deaths – 520 in the control arm and 130 in the screening arm – the power of the study would be 89%. Hence, we aim at a final sample size of at least 111,000 men to ensure adequate statistical power and precision at the final analysis.

Randomization and screening intervals

All eligible men will be randomly allocated to screening and control arms in a 1:3 ratio. Within the screening arm, re-screening interval is adapted by the baseline PSA:

- Men with initial PSA≥3 ng/ml are re-invited every two years,
- Men with PSA 1.5–2.99 ng/ml every four years, and
- Men with PSA<1.5 ng/ml after six years.

By the time of writing this plan, we have randomized 61,193 men with 15,299 allocated to the screening arm and 45,894 to the control arm. Analyses will compare the entire screening arm, regardless of the actual screening attendance and interval employed, to the control arm, unless otherwise specified.

Randomization list consists of batches of randomized men. The list is generated centrally by a designated study biostatistician at the coordinating unit, who maintains the documentation including program codes and the resulting lists include information of randomization dates, personal identification numbers (linkable to study ID number) and the arm allocated. Randomization lists are only shared confidentially to study personnel if needed for study conduct.

For peer review only

Screening procedures

At every screening attendance, three consecutive tests are conducted in a stepwise manner before biopsy:

1. All participating men give a blood sample for determination of PSA at a local laboratory.
2. If the PSA is 3 ng/ml or higher, a four-kallikrein panel is analyzed from a second vial of plasma from the initial draw using an algorithm incorporating four proteins (total PSA, free PSA, intact PSA and human kallikrein-2) and age. The result is expressed as probability of a clinically significant PCa.
3. Men with both PSA≥3 and kallikrein score≥7.5% are referred to MRI. T2-weighted, diffusion-weighted and dynamic contrast-enhanced imaging is employed in accordance with the European Society for Urogenital Radiology guideline (de Rooij 2020). The findings are classified according to the Prostate Imaging Reporting and Data System (PI-RADS v2.1), which is a 5-point scale to combine the MRI findings and indicate the likelihood of a significant cancer. Scores of 3–5 indicate at least a suspect finding warranting directed biopsy.

Only targeted biopsies are employed, with 2–4 cores per region of interest depending on the size. Only screen-positive men with negative MRI but PSA density >0.15 undergo systematic biopsy as a safety measure (to avoid missing clinically significant cancers). Similar fusion-guided biopsy systems are used at the two trial sites and evaluated by experienced uropathologists using standardized procedures.

A random sample of screen-negative (on test steps 1 and 2) men are also invited to prostate MRI and asked to give blood, urine and stool samples in order to serve as a control group to estimate frequency of suspicious MRI findings in the general population, and as a reference group in analyses of biological samples.

Protocol deviations

A tabular presentation of different types of protocol deviations along with their frequencies and percentages will be presented. Any protocol deviations detected after randomization will be carefully documented. Among them, men later found out not to have met the eligibility criteria at the date of randomization can be excluded from the analysis (post-randomization exclusions).

In the case of major protocol violations affecting a substantial proportion of men, separate per protocol analyses will be conducted to support the main analyses. In the screening arm, incomplete attendance, or compliance with the screening procedures is likely to occur. In the control arm, we will obtain data on contamination, i.e., mostly self-initiated PSA testing.

When considering unforeseen lack of compliance with the protocol, all means to ensure objectivity in the exclusion principles from per protocol analyses will be taken. Participants in both arms will be considered according to the same principles. Protocol deviations not related to the screening procedures are expected to appear in approximately 1:3 ratio for the arms. Obvious deviation from this ratio would be reported and interpreted as a potential source of bias.

For peer review only

Blinding

Blinding in the conventional sense is not applicable: men are aware of being invited to screening. Hence, this is an open trial with screening and control arms.

Concrete measures to prevent bias, if any, from the awareness of the trial arm were nevertheless taken: (i) the control arm is blind to the fact that they are part of the trial; (ii) allocation concealment is ensured by the centralized randomization procedure preventing foreknowledge of upcoming arm allocation; and (iii) communication to the general public on trial is kept to the minimum to prevent contamination (e.g. by self-initiated PSA testing) among men in the control arm.

In addition, we underline that the primary outcome of the study, PCa death, is an objective outcome. The possibility of bias in its evaluation only relates to the assessment of the cause of death. The death certificates are filled by physicians with no involvement in the trial and can be assumed to be independent of trial arm, especially as deaths from prostate cancer are likely to occur years after the diagnosis and hence unaffected by detection through screening or other means. Importantly, a previous study within the ERSPC trial has shown that the cause-of-death data provided by Statistics Finland agreed almost perfectly with the assessment of a blinded expert panel in the Finnish center of the trial and was independent of the trial arm (Mäkinen et al. 2008, Kilpeläinen et al. 2016).

Data collection process

Table 1 summarizes the stages of the data collection process, targeted participants, and information and samples obtained.

Table 1. Data collection process of the ProScreen trial.

Process stage	Target population	Information collected	Samples collected
**Baseline**	Participants	Family history<br>Previous PSA and Bx<br>Generic QoL/utility (15D, EQ5D)<br>Out-of-pocket costs<br>PSA and four-kallikrein panel	Plasma<br>Serum<br>Whole blood
**MRI**	Men with PSA >3 ng/ml and kallikrein score >7.5%	PIRADS score	Digital image
**Biopsy**	Screen-positive men	Post-biopsy symptoms (0, 30 days)<br><br>Targeted fusion biopsies: number of ROIs, number of biopsies, length of samples,<br><br>Systemic biopsies: Biopsy length, cancer length and Gleason score per sample, total length of samples, total length of cancer, portion of cancer, global Gleason score, portion of Gleason 4 or 5, perineural invasion	Urine<br>Stool<br>RNA, DNA<br>Cancer tissue and prostate tissue<br>Plasma<br>Serum<br>Whole blood
**Cancer**	Men with	Disease-specific QoL (EPIC-26, MAX-PC)	

For peer review only

**diagnosis**	prostate cancer	Generic Qol/utility (EQ5D, 15D), out-of-pocket costs<br><br>Gleason/ISUP grade group, number of positive cores, length of cancer, treatment, TNM stage	

Study outcomes and other relevant variables

The primary outcome of the trial is death from prostate cancer. Causes of death will be obtained from the Statistics Finland database and the underlying causes of death will be considered when evaluating if the man died from PCa or from other causes. Cancer cases in the entire trial population including the control arm and non-participants in the screening arm are identified from pathology databases of the two hospitals and through linkages to the Finnish Cancer Registry using the unique PID assigned to all Finnish residents to ensure complete coverage and avoid duplicates (double count).

Secondary outcomes are:

- Diagnosis of prostate cancer (divided into clinically significant and insignificant)
- Progression to advanced prostate cancer (biochemical relapse or progression to metastatic)
- Death due to any cause
- Cost-effectiveness of screening

Adverse outcome variables to monitor screening-related harms are:

- Overdiagnosis of clinically insignificant prostate cancer
- Quality of life impacts of screening and quality of life among men with PCa (EPIC26 instrument)
- Prostate cancer-related anxiety (MAX-PC questionnaire)
- Complications from biopsy (PRECISION questionnaire)

Statistical analysis

The main analyses will rely on the intention to screen (ITS) principle and will include all randomized men in the two trial arms who were alive and eligible (free of prostate cancer) at the date of randomization. Those men who became ineligible between the date of randomization and first screening invitation will remain in the ITS analysis set.

Two-sided statistical tests will be used, and the overall significance level will be set at 5%. Corresponding p-values will be accompanied with estimates of differences and their 95% confidence intervals.

Analysis of the primary outcome

The primary outcome of the trial is death from prostate cancer. This is a superiority trial regarding the primary outcome and the comparisons between trial arms will be analyzed and presented on this basis.

Those men who survived will be considered as right-censored observations at the time passed between the time of analysis and time of randomization period. Those men who were lost to

For peer review only - http://bmjopen.bmj.com/site/about/guidelines.xhtml

follow-up (e.g., due to emigration) will be considered as censored at that particular time (e.g., at emigration). Time to death, defined as the difference between the date of death and date of randomization, will be used as the event time for the analysis.

To evaluate differences between screening and control arms in prostate cancer specific mortality, Gray's test (Gray 1988) for testing the null hypothesis of equality of cumulative incidence functions will be used. This test differs from the commonly used logrank test in how competing risks of death are treated and is based on the subdistribution hazard of prostate cancer cause of death.

The test will be complemented by reporting the number of PCa deaths, number of men at risk and estimated cumulative incidence functions for each trial arm over follow-up time. The arms will be compared in absolute risks (number needed to invite i.e., the inverse of the risk difference and number needed to diagnose per averted prostate cancer death, i.e., the ratio of excess incidence to mortality reduction), as well as and relative measures of effect (hazard ratios). Descriptive summaries will also be presented by trial centers, age group at randomization.

Secondary analyses of the primary outcome

Fine-Gray model for the subdistribution hazard will be used to conduct analyses adjusted for background factors. Outcomes will be compared between age groups and trial centers, and in case of differences, analyses to control for trial center and for age at randomization (categorized as 50–54, 55–59, 60–65 years) will be conducted.

Per protocol analyses excluding men with substantial protocol deviations, such as repeated non-attendance or ineligibility before invitation to screening (with pseudo invitation dates for the control arm), will be conducted if considered pertinent. Additional analyses to correct for contamination and non-compliance, i.e., estimation of efficacy, will be taken by best practices methods at the time of the analyses (e.g., Cuzick et al. 1997).

Descriptive analyses to study effect heterogeneity by center and age group will be performed to complement these analyses. Additional analyses requested by external reviewers or editors in peer-review processes will also be done.

Analysis of secondary outcomes

Diagnosis of prostate cancer

The analysis of cumulative incidence of PCa by disease aggressiveness intends to assess screening impact on detection of clinically significant PCa (representing potential benefit through early treatment) and clinically insignificant PCa (indicating overdiagnosis). The intention is to assess the extent of detection of clinically significant PCa by screening relative to the control arm, and extent of overdiagnosis relative to the control arm. This will inform about the degree of accomplishing rationale of the trial, i.e., detection of aggressive cases at least similar to that in PSA-based screening, while substantially decreasing the yield of low-risk cases. As screening advances the time of diagnosis by several years (lead time), cumulative incidence will be used as the indicator of risk.

Disease aggressiveness will be defined by the International Society for Urological Pathology (ISUP) Gleason grade group. The analyses will be conducted separately for the detection of clinically significant (Gleason 7+ or ISUP 2+) and clinically insignificant (Gleason <7 or ISUP 1) PCa. In secondary analyses, alternative criteria for csPCa will also be employed

For peer review only - http://bmjopen.bmj.com/site/about/guidelines.xhtml

including ISUP 3+ (Gleason 4+3 or higher), maximum length of cancer tissue in biopsy and number of biopsy cores with cancer.

Risk differences and ratios will be used to infer screening benefits and overdiagnosis compared to the control arm. Besides cumulative incidence, the ratio of aggressive to non-aggressive cases (or proportion of aggressive cancers out of all PCa) will also be reported.

Cumulative incidence for both outcomes will be estimated by trial arm. The overall PCa incidence combines screening benefits and harms and is thus regarded of minor importance in the interpretation of screening impact. Tabular presentations of age at diagnosis, disease stage and grade at diagnosis will be presented.

Both intention to screen (by allocation) and per protocol (screening participants and non-participants) analyses will be conducted for each screening round. For screening participants, screen-detected and interval cases will be reported separately, and screen-detected cases will be broken down by those detected in targeted biopsies of MRI-positive lesions (screening protocol evaluated) and systematic biopsies in screen-negative men with PSA density >0.15 (safety measure to avoid missing clinically significant cases). Any cases detected in a random sample of screen-negative men invited to MRI (analyses to assess underlying prevalence of prostate cancer) will also be reported separately. Analyses to evaluate an optimized screening algorithm will include exclusion of cases with PI-RADS score 3 and kallikrein score calculated also incorporating information on previous biopsies (ignored in the main analysis), as well as using higher cut-off values for PSA and the kallikrein score.

Advanced prostate cancer

The analysis of advanced prostate cancer will compare the cumulative incidence of cancer progression, including metastasis and/or biochemical relapse developing after diagnosis and primary treatment, between the screening and control arms. The purpose of the analysis is to evaluate differences between the arms in the risk of developing a potentially lethal, advanced PCa.

The origin of the analysis will be the time of randomization. Cumulative incidence rates will be estimated by the Kaplan-Meier method, and differences between trial arms will be estimated by Cox regression models adjusted by age at diagnosis.

Death due to any cause

The analysis of all-cause mortality aims to show that the trials arms are comparable with each other and the general male population in Finland. These analyses will not inform about the effectiveness of screening. Cumulative survival and mortality rates will be estimated by the Kaplan-Meier method, from time of randomization, displayed with frequencies of events and men at risk by trial arm, and by age at randomization.

This analysis will focus on the intention to screen analysis set.

Cost-effectiveness

A cost-effectiveness analysis will be performed, incorporating cost data for both out-of-pocket estimated from surveys and service cost data collected from health care providers, as well mortality results (ITS analysis) and utilities based on repeated surveys with 15D and EQ5D instruments (on a random sample of participants). The comparator is no active screening, here represented by the control arm. The main outcome is the incremental cost-effectiveness ratio in terms of costs per quality-adjusted life-year.

For peer review only

A preliminary and exploratory cost effectiveness study can be conducted after the last of the follow-up surveys have been returned, approximately at 3 years after the randomization of the last man into the trial. We plan to undertake a full cost-effectiveness analysis around the time when the evidence on the effectiveness of screening regarding primary outcome has been obtained; this will most likely be near to the analysis at 15 years.

Quality of life

These analyses aim to evaluate the short-term and long-term impacts of screening on generic quality of life as well as disease-specific quality of life among men with PCa. Two disease-specific questionnaires, EPIC26 instrument and MAX-PC questionnaires will be used to measure quality of life at 0, 6, 12, and 24 months from PCa diagnosis in both trial arms.

Standard scoring of the EPIC26 instrument will be used. Summary statistics of the five key domains over time and by trial arm will be calculated to assess changes in quality of life of men with PCa from diagnosis onwards. Summary and domain-grouped scores will be analyzed using applications of linear models (or their nonparametric counterparts, if needed) for repeated measures to evaluate differences in quality of life between the arms following PCa diagnosis.

Prostate cancer related anxiety is measured with the Memorial Anxiety Scale for Prostate Cancer (MAX-PC) questionnaire (Roth et al. 2003). Results will be presented as frequencies and percentages for total and subscale scores by trial arm.

Generic quality of life and utilities are evaluated using the 15D and EQ5D instruments as described in the cost-effectiveness section.

Analysis of adverse outcomes

In addition to detection of low-risk disease by screening as an indicator of overdiagnosis, adverse outcomes mainly relate to the harms due to biopsies. Adverse effects of prostate biopsy are monitored using the questionnaire developed for the PRECISION trial covering pain and other symptoms immediately after biopsy and at 30 days following biopsy. The number of biopsies, as well as the number (%) and type of complications among those with biopsies will be reported.

Interim analyses and data monitoring

The first analysis of PCa mortality will be conducted at 10 years and the final analysis at 15 years (i.e. at median follow-up time of 10 or 15 years). As we do not intend to stop the trial at 10 years, these interim analyses will be considered as preliminary information. Interim analyses at 10 years will include also analyses of shorter-term benefits.

To control the overall type I error rate (5%) of the trial, we will employ the O'Brien-Fleming rule for alpha spending function. We set the amount of information at 0.5 at 10 years based on the expected numbers of PCa deaths. Thus, by implementation of the O'Brien-Fleming algorithm, the resulting significance level at 10-year interim analysis will be 0.0056, and at the 15-year final analysis 0.0444.

Analyses of secondary endpoints informing about the intermediate outcomes of process indicators including participation, cancer detection, validity and diagnostic performance of the tests in the entire population and subgroups (screened men, non-participants, men in the control arm) will be carried out at regular intervals, as sufficient data become available for evaluation. These will inform about potential need to modify the procedures. Side studies

For peer review only

using the samples collected will be carried out to identify new indicators of prostate cancer risk and prognosis.

An independent data monitoring committee (DMC) oversees the trial conduct, and its main task is to ensure safety of the participants. Safety in this context means that screening or screening procedures should not lead to unacceptable disadvantage for the participants in the light of screening benefits. This could take place if the screening intervention had materially worse performance in detecting clinically relevant prostate cancer than anticipated, or substantially higher level of overdiagnosis. The DMC is given a report of the screening results initially every six months and after the first year every 12 months. The DMC can also request any additional information they regard as pertinent to their task. In case of concern, the DMC can recommend discontinuation of the trial; in practice that would mean stopping recruitment and discontinuation of further screening procedures. In addition, they have a mandate to suggest modifications to the trial protocol.

Handling of missing data

Extent of missing data will be described, for example, by presenting the number of individuals with missing values per variable.

For outcome variables relying on dates – dates of randomization, censoring, diagnosis or death – incomplete dates will be imputed by 15 (in the case that the day variable was missing, but known month and year), and by 30/6 (in the case that only the year was known).

In case a substantial proportion of men (at least 5%) have missing data on one or more variable needed for the effectiveness analysis in question, multiple imputation methods will be used to demonstrate the robustness of findings (Little *et al.* 2012). Imputation models will include outcome variables and trial arm in addition to all variables relevant to the particular analysis. Final estimates will be derived by combining estimates and their standard errors across data sets using Rubin's rules.

Data management and quality assurance

RedCap database application is used for data management in the trial, covering all major data types from questionnaires and lab results to MRI findings, diagnoses and causes of death. RedCap allows access defined by two-factor authentication (2FA) and flexible definition of user-specific functions and rights.

In REDCap, variable specific parameters and predetermined options are used to prevent entering invalid data (e.g. predefined values and acceptable ranges). All data is verified from the original data source and monitored monthly. Until the verification, data is saved as incomplete or unverified. Lead times between screening tests are monitored every 6-8 weeks. For the laboratory work (including sampling, processing, and storing) each task has a protocol shared by the study centers. Any deviations from the sample specific protocol are documented.

Conclusion

This statistical analysis plan lays out the plans for outcomes of the trial, including the definitions of important outcomes, analysis principles and interpretation, methods for primary analysis, pre-specified subgroup analysis, and secondary analysis.

For peer review only

References

Ahmed HU, El-Shater Bosaily A, Brown LC, *et al*. Diagnostic accuracy of multi-parametric MRI and TRUS biopsy in prostate cancer (PROMIS): a paired validating confirmatory study. *Lancet* 2017; 389:815-822. doi: 10.1016/S0140-6736(16)32401-1. PMID: 28110982.

Auvinen A, Rannikko A, Taari K, *et al*. A randomized trial of early detection of clinically significant prostate cancer (ProScreen): study design and rationale. *Eur J Epidemiol* 2017; 32:521-527. doi: 10.1007/s10654-017-0292-5. PMID: 28762124.

Culp MB, Soerjomataram I, Efstathiou JA, *et al.* Recent global patterns in prostate cancer incidence and mortality rates. *Eur Urol* 2020; 77:38-52

Cuzick J, Edwards R, Segnan N. Adjusting for non-compliance and contamination in randomized clinical trials. *Stat Med* 1997; 16:1017-29. doi: 10.1002/(sici)1097-0258(19970515)16:9<1017::aid-sim508>3.0.co;2-v. Erratum in: *Stat Med* 2007;26:3821. PMID: 9160496.

Eklund M, Jäderling F, Discacciati A, *et al*. MRI-Targeted or Standard Biopsy in Prostate Cancer Screening. *N Engl J Med* 2021; 385:908-920. doi: 10.1056/NEJMoa2100852. PMID: 34237810.

Fenton JJ, Weyrich MS, Durbin S, *et al*. Prostate-specific antigen-based screening for prostate cancer. *JAMA* 2018; 319:1914-31.

Gamble C, Krishan A, Stocken D, *et al*.. Guidelines for the content of statistical analysis plans in clinical trials. *JAMA* 2017; 318:2337–43. https://doi.org/10.1001/jama.2017.18556.

Gray RJ: A class of K-sample tests for comparing the cumulative incidence of a competing risk. *Ann Stat* 1988; 16:1141-1154.

Hugosson J, Roobol MJ, Månsson M, *et al*. 16-year follow-up of the European Randomised Study of Screening for Prostate Cancer (ERSPC). *Eur Urol* 2019; 76:43-51.

Hugosson J, Månsson M, Wallström J, *et al*. Prostate Cancer Screening with PSA and MRI Followed by Targeted Biopsy Only. *N Engl J Med*. 2022; 387:2126-2137. doi: 10.1056/NEJMoa2209454. PMID: 36477032.

Ilic D, Djulbegovic M, Jung JH, *et al*. Prostate cancer screening with prostate-specific antigen (PSA) test: a systematic review and meta-analysis. *BMJ* 2018; 362:k3519. doi: 10.1136/bmj.k3519. PMID: 30185521.

Kilpeläinen TP, Mäkinen T, Karhunen PJ, *et al*. Estimating bias in causes of death ascertainment in the Finnish Randomized Study of Screening for Prostate Cancer. *Cancer Epidemiol* 2016; 45:1-5. doi: 10.1016/j.canep.2016.08.022. PMID: 27636505.

Little RJ, D'Agostino R, Cohen ML, *et al*. The prevention and treatment of missing data in clinical trials. *N Engl J Med* 2012; 367:1355-60. doi: 10.1056/NEJMsr1203730. PMID: 23034025.

Mäkinen T, Karhunen P, Aro J, *et al*. Assessment of causes of death in a prostate cancer screening trial. Int J Cancer 2008; 122: 413-417. https://doi.org/10.1002/ijc.23126

For peer review only - http://bmjopen.bmj.com/site/about/guidelines.xhtml

Paschen U, Sturz S, Fleer D, *et al*. Assessment of prostate-specific antigen screening. *BJU Int* 2022;129:280-289

Pinsky PF, Miller E, Prorok P, *et al*. Extended follow-up for prostate cancer incidence and mortality among participants in the PLCO screening trial. *BJU Int* 2019;123:854-860

Schoots IG, Roobol MJ, Nieboer D, *et al*. Magnetic resonance imaging-targeted biopsy may enhance the diagnostic accuracy of significant prostate cancer detection compared to standard transrectal ultrasound-guided biopsy: a systematic review and meta-analysis. *Eur Urol* 2015; 68:438-50. doi: 10.1016/j.eururo.2014.11.037. PMID: 25480312.

Rannikko A, Leht M, Mirtti T, *et al*. Population-based randomized trial of screening for clinically significant prostate cancer ProScreen: a pilot study. *BJU Int* 2022; 130:193-199. doi: 10.1111/bju.15683. PMID: 34958531.

de Rooij M, Israël B, Tummers M, *et al*. ESUR/ESUI consensus statements on multi-parametric MRI for the detection of clinically significant prostate cancer: quality requirements for image acquisition, interpretation and radiologists' training. *Eur Radiol* 2020; 30:5404-5416. doi: 10.1007/s00330-020-06929-z. PMID: 32424596.

Roth AJ, Rosenfeld, B, Kornblith AB, *et al*. The memorial anxiety scale for prostate cancer. *Cancer* 2003; 97:2910-18

Statistics Finland's StatFin online service. Available at: https://statfin.stat.fi/PXWeb/pxweb/en/StatFin/ (Accessed 9 June 2021).

Schoenfeld DA. Sample-size formula for the proportional-hazards regression model. *Biometrics* 1983; 39:499-503.

Authors' contributions

All authors approved the final version of this manuscript. Study concept and design: AA, AR, JN, JR, KN, TK, TT; drafting of the manuscript: AA, JN; critical revision of the manuscript for important intellectual content: AA, AR, JN, JR, KN, TK, TT.

Funding statement

This work was supported by the Academy of Finland (grant number 311336) the Finnish Cancer Foundation, the Jane and Aatos Erkko foundation, Competitive State Research Funding administered by Tampere University Hospital (grant number 9V02), and Päivikki and Sakari Sohlberg Foundation.

For peer review only

Protocol summary and statistical analysis plan for the randomized trial of early detection of clinically significant prostate cancer (ProScreen)

Journal:	*BMJ Open*
Manuscript ID	bmjopen-2023-075595.R1
Article Type:	Protocol
Date Submitted by the Author:	07-Dec-2023
Complete List of Authors:	Nevalainen, Jaakko; Tampere University<br>Raitanen, Jani; Tampere University, Faculty of Social Sciences (Health Sciences) and Gerontology Research Center<br>Natunen, Kari; Tampere University<br>Kilpelainen, Tuomas; University of Helsinki; Helsinki University Central Hospital<br>Rannikko, Antti; University of Helsinki; Helsinki University Central Hospital<br>Tammela, Teuvo; Tampere University; Tampere University Hospital<br>Auvinen, Anssi; Tampere University
**Primary Subject Heading**:	Urology
Secondary Subject Heading:	Epidemiology, Oncology, Public health
Keywords:	Mass Screening, Prostatic Neoplasms, Randomized Controlled Trial, STATISTICS & RESEARCH METHODS

SCHOLARONE™ Manuscripts

Title: Protocol summary and statistical analysis plan for the randomized trial of early detection of clinically significant prostate cancer (ProScreen)

Authors: Jaakko Nevalainen[1,*], Jani Raitanen[1,2], Kari Natunen[1], Tuomas Kilpeläinen[3,4], Antti Rannikko[3,4], Teuvo Tammela[5], Anssi Auvinen[1], and the ProScreen Trial Team

Affiliations

1. Unit of Health Sciences, Faculty of Social Sciences, Tampere University, Tampere, Finland
2. The UKK Institute for Health Promotion Research, Tampere, Finland
3. Department of Urology, University of Helsinki and Helsinki University Hospital, Helsinki, Finland
4. Research Program in Systems Oncology, Faculty of Medicine, University of Helsinki, Helsinki, Finland
5. Tampere University Hospital, Department of Urology and Tampere University, Faculty of Medicine and Health Technology, Tampere, Finland

* = Corresponding author (email: jaakko.nevalainen@tuni.fi)

ABSTRACT

Introduction: Evidence on the effectiveness of prostate cancer screening based on prostate-specific antigen is inconclusive and suggests a questionable balance between benefits and harms due to overdiagnosis. However, diagnostic accuracy studies have shown that detection of clinically insignificant prostate cancer can be reduced by magnetic resonance imaging combined with targeted biopsies.

The aim of the paper is to describe the analysis of the ProScreen randomized trial to assess the performance of the novel screening algorithm in terms of the primary outcome, prostate cancer mortality, and secondary outcomes as intermediate indicators of screening benefits and harms of screening.

Methods: The trial aims to recruit at least 111,000 men to achieve sufficient statistical power for the primary outcome. Men will be allocated in a 1:3 ratio to the screening and control arms. Interim analysis is planned at 10 years of follow-up, and the final analysis at 15 years. Difference between the trial arms in prostate cancer mortality will be assessed by Gray's test using intention to screen analysis of randomized men. Secondary outcomes will be the incidence of prostate cancer by disease aggressiveness, progression to advanced prostate cancer, death due to any cause and cost-effectiveness of screening.

Ethics and dissemination: The trial protocol was reviewed by the ethical committee of the Helsinki University Hospital (HUS 2910/2017). Results will be disseminated through publications in international peer-reviewed journals and at scientific meetings.

Trial Registration: NCT03423303

Keywords: effectiveness; prostate cancer screening; randomized trial; screening algorithm

For peer review only

STRENGTHS AND LIMITATIONS

- This population-based, randomized multicenter trial targeting at recruiting 111,000 men will provide high quality evidence on the effectiveness of a novel screening strategy for prostate cancer mortality
- Broad eligibility criteria and pragmatic approach embedded in normal clinical practice enhances the external validity of the trial and provide evidence applicable to decision making in public health and health care
- Challenges for the trial include the maintenance of high compliance to screening and the extent of opportunistic PSA testing in the population

For peer review only

Introduction

Prostate cancer is the most common cancer in men in many industrialized countries and causes substantial mortality [1]. Screening based on blood prostate-specific antigen (PSA) has been shown to decrease prostate cancer mortality, but the evidence from randomized trials is not conclusive [2, 3]. Systematic reviews of randomized controlled trials have concluded that PSA screening may at best lower prostate cancer mortality, but not all-cause mortality. However, the balance between benefits and harms was regarded as problematic due to frequent overdiagnosis, and complications from biopsies and overtreatment [4, 5, 6].

Several studies have shown that detection of clinically insignificant prostate cancer can be reduced by magnetic resonance imaging (MRI) combined with targeted biopsies of the suspect foci, instead of systematic biopsies of the entire prostate [7, 8]. However, previous studies have mostly focused on the diagnostic performance, i.e., cancer detection at a single evaluation. A hybrid screening/diagnostic study and a screening trial using MRI were recently published [9, 10].

Here we describe the analysis of the ProScreen randomized screening trial to assess the performance of a novel screening algorithm in terms of the primary outcome, prostate cancer mortality, and secondary outcomes, used as intermediate indicators of benefits and harms of screening. Following good statistical practice, this statistical analysis plan (version: 1.1) was finalized prior to completion of recruitment and short-term follow-up data collection. It was written following the guidelines provided in Gamble *et al.* [11] as applicable. Any unforeseen deviations from the plan will be described and justified carefully in the respective reports.

Trial overview

Trial design

The ProScreen trial is a population-based, randomized multicenter trial that investigates the effectiveness of a novel screening strategy combining PSA, a four-kallikrein panel, and MRI on prostate cancer (PCa) mortality over a 15-year period from randomization [12]. The rationale is to minimize detection of clinically insignificant cancers, while maintaining a high sensitivity for aggressive cancers in order to reduce overdiagnosis without compromising mortality benefits. An interim analysis of PCa mortality is planned at 10 years of follow-up.

Ethics and Dissemination

On 15 January 2018, the trial was registered at clinicaltrials.gov (NCT03423303). The ethical committee of Helsinki University Hospital reviewed the protocol (tracking no. 2910/2017). Permissions to collect data from health care registers was obtained from Finnish Institute for Health and Welfare (before the era of FinData, Dnro THL/676/5.05.00/2018). A written informed consent is provided by each participant in the screening arm. Results will be disseminated through publications in international peer-reviewed journals and at scientific meetings.

Recruitment started in October 2018 and is still ongoing.

Patient and public involvement

Patients or the public were not involved in the design, or conduct, or reporting, or dissemination plans of our research.

Study population

All men aged 50–63 years (at the time of sampling of the trial population) with Finnish or Swedish as mother tongue residing in the trial municipalities constitute the trial population.

For peer review only

Men with a prevalent prostate cancer will be identified through the Finnish Cancer Registry or hospital pathology databases and excluded.

We have identified for the trial the entire target population from the Digital and Population Data Services Agency, comprehensively without any sampling. The initial trial population consists of men residing in Helsinki and Tampere.

Currently, we are increasing the sample size by recruiting men also in the other municipalities within the Helsinki and Tampere metropolitan areas (Vantaa, Espoo, and Kauniainen, as well as Nokia, Lempäälä, Pirkkala, Ylöjärvi, and Kangasala with a total of 57,000 men in the target age group). The target population covers comprehensively all eligible men in the municipalities, both in the original Helsinki and Tampere areas and the new municipalities.

Sample size

We estimated that we could find 110,000–120,000 men in the target age group based on the population projections from 2020 to 2034 from the ten municipalities [13]. We requested the overall number of deaths and the number of PCa deaths from Statistics Finland by age group from 1990 to 2019. The proportion of PCa deaths had barely changed at all during the 30-year period and hence, we based our sample size calculation on these figures. With a 1:3 random allocation to the screening arm relative to the control arm, we expected that we would follow-up on 82 500 men in the control arm. Based on population statistics and causes of death registries, for the first ten years of the trial, we estimated PCa mortality to be 50 deaths per 100,000 person-years. For the entire 15 years follow-up, our estimate was 67 deaths per 100,000 person-years. Given deaths due to other causes, we thus expected to include at least 240 and 520 PCa deaths in the control arm during the first 10 and 15 years of the trial, respectively.

Considering that a PSA-based screening could result into a 20% reduction in PCa mortality [2], and that the addition of MRI and 4K to the screening protocol could improve the ability to detect clinically significant PCa [14], we assumed a relative hazard of 0.75 for the screening relative to the control arm. Schoenfeld's formula indicates that an 80% power would be reached by a total of 506 PCa deaths [15] with type I error rate set at 5%. Assuming a total of 650 PCa deaths – 520 in the control arm and 130 in the screening arm – the power of the study would be 89%. Hence, we aim at a final sample size of at least 111,000 men to ensure adequate statistical power and precision at the final analysis.

Randomization and screening intervals

All eligible men will be randomly allocated to screening and control arms in a 1:3 ratio. The rationale for the unequal randomization arose from the logistics in the conduct of the trial: we first estimated the maximum number of men that could be screened with existing resources. Given that the size of the control arm that does not receive any intervention has hardly any resource implications, we decided on an allocation ratio that would yield a manageable size of the intervention arm. Hence, the underlying rationale was to fix the size of the screening and maximize the size of the control arm to optimize the power of the study given the context (source population).

Within the screening arm, re-screening interval is adapted by the baseline PSA:

- Men with initial PSA≥3 ng/ml are re-invited every two years,
- Men with PSA 1.5–2.99 ng/ml every four years, and

For peer review only

- Men with PSA<1.5 ng/ml after six years.

The control arm is unaware of being part of the trial, as they are not contacted or subject to any trial procedures. At prostate cancer diagnosis, the aim is that all men in the control arm will also answer questionnaires, but otherwise they are treated according to standard clinical practice according to the well-established national treatment guidelines throughout the trial.

By the time of writing this plan, we have randomized 61,193 men with 15,299 allocated to the screening arm and 45,894 to the control arm. Analyses will compare the entire screening arm, regardless of the actual screening attendance and interval employed, to the control arm, unless otherwise specified.

Randomization list consists of batches of randomized men, *i.e.*, randomization list is generated in parts to ensure that the time between randomization and the invitation to the trial would not be excessive. Stratified randomization was not considered necessary. The list is generated centrally by a designated study biostatistician at the coordinating unit, who maintains the documentation including program codes and the resulting lists include information of randomization dates, personal identification numbers (linkable to study ID number) and the arm allocated. Randomization lists are only shared confidentially to study personnel if needed for study conduct.

Screening procedures

At every screening attendance, three consecutive tests are conducted in a stepwise manner before biopsy:

1. All participating men give a blood sample for determination of PSA at a local laboratory.
2. If the PSA is 3 ng/ml or higher, a four-kallikrein panel is analyzed from a second vial of plasma from the initial draw using an algorithm incorporating four proteins (total PSA, free PSA, intact PSA and human kallikrein-2) and age. The result is expressed as probability of a clinically significant PCa.
3. Men with both PSA≥3 and kallikrein score≥7.5% are referred to MRI. T2-weighted, diffusion-weighted and dynamic contrast-enhanced imaging is employed in accordance with the European Society for Urogenital Radiology guideline [16]. The findings are classified according to the Prostate Imaging Reporting and Data System (PI-RADS v2.1), which is a 5-point scale to combine the MRI findings and indicate the likelihood of a significant cancer. Scores of 3–5 indicate at least a suspect finding warranting directed biopsy.

Only targeted biopsies are employed, with 2–4 cores per region of interest depending on the size. Only screen-positive men with negative MRI but PSA density >0.15 undergo systematic biopsy as a safety measure (to avoid missing clinically significant cancers). Similar fusion-guided biopsy systems are used at the two trial sites and evaluated by experienced uropathologists using standardized procedures.

A random sample of screen-negative (on test steps 1 and 2) men are also invited to prostate MRI and asked to give blood, urine and stool samples in order to serve as a control group to estimate frequency of suspicious MRI findings in the general population, and as a reference group in analyses of biological samples.

For peer review only

Protocol deviations

A tabular presentation of different types of protocol deviations along with their frequencies and percentages will be presented. Any protocol deviations detected after randomization will be carefully documented. Among them, men later found out not to have met the eligibility criteria at the date of randomization will be excluded from the analysis (post-randomization exclusions).

In the case of major protocol violations affecting a substantial proportion of men, separate per protocol analyses will be conducted to support the main analyses. In the screening arm, incomplete attendance, or compliance with the screening procedures is likely to occur. In the control arm, we will obtain data on contamination, i.e., mostly self-initiated PSA testing.

When considering unforeseen lack of compliance with the protocol, all means to ensure objectivity in the exclusion principles from per protocol analyses will be taken. Participants in both arms will be considered according to the same principles. Protocol deviations not related to the screening procedures are expected to appear in approximately 1:3 ratio for the arms. Obvious deviation from this ratio would be reported and interpreted as a potential source of bias.

Blinding

Blinding in the conventional sense is not applicable: men are aware of being invited to screening. Hence, this is an open trial with screening and control arms.

Concrete measures to prevent bias, if any, from the awareness of the trial arm were nevertheless taken: (i) the control arm is blind to the fact that they are part of the trial; (ii) allocation concealment is ensured by the centralized randomization procedure preventing foreknowledge of upcoming arm allocation; and (iii) communication to the general public on trial is kept to the minimum to prevent contamination (e.g. by self-initiated PSA testing) among men in the control arm.

In addition, we underline that the primary outcome of the study, PCa death, is an objective outcome. The possibility of bias in its evaluation only relates to the assessment of the cause of death. The death certificates are filled by physicians with no involvement in the trial and can be assumed to be independent of trial arm, especially as deaths from prostate cancer are likely to occur years after the diagnosis and hence unaffected by detection through screening or other means. Importantly, a previous study within the ERSPC trial has shown that the cause-of-death data provided by Statistics Finland agreed almost perfectly with the assessment of a blinded expert panel in the Finnish center of the trial and was independent of the trial arm [17, 18].

Data collection process

Table 1 summarizes the stages of the data collection process, targeted participants, and information and samples obtained.

For peer review only

Table 1. Data collection process of the ProScreen trial.

Process stage	Target population	Information collected	Samples collected
**Baseline**	Participants	Family history<br>Previous PSA and Bx<br>Generic QoL/utility (15D, EQ5D)<br>Out-of-pocket costs<br>PSA and four-kallikrein panel	Plasma<br>Serum<br>Whole blood
**MRI**	Men with PSA >3 ng/ml and kallikrein score >7.5%	PIRADS score	Digital image
**Biopsy**	Screen-positive men	Post-biopsy symptoms (0, 30 days)<br><br>Targeted fusion biopsies: number of ROIs, number of biopsies, length of samples,<br><br>Systemic biopsies: Biopsy length, cancer length and Gleason score per sample, total length of samples, total length of cancer, portion of cancer, global Gleason score, portion of Gleason 4 or 5, perineural invasion	Urine<br>Stool<br>RNA, DNA<br>Cancer tissue and prostate tissue<br>Plasma<br>Serum<br>Whole blood
**Cancer diagnosis**	Men with prostate cancer	Disease-specific QoL (EPIC-26, MAX-PC)<br><br>Generic Qol/utility (EQ5D, 15D), out-of-pocket costs<br><br>Gleason/ISUP grade group, number of positive cores, length of cancer, treatment, TNM stage	

Study outcomes and other relevant variables

The primary outcome of the trial is death from prostate cancer. Causes of death will be obtained from the Statistics Finland database and the underlying causes of death will be considered when evaluating if the man died from PCa or from other causes. Cancer cases in the entire trial population including the control arm and non-participants in the screening arm are identified from pathology databases of the two hospitals and through linkages to the Finnish Cancer Registry using the unique PID assigned to all Finnish residents to ensure complete coverage and avoid duplicates (double count).

Secondary outcomes are:

- Diagnosis of prostate cancer (divided into clinically significant and insignificant)
- Progression to advanced prostate cancer (biochemical relapse or progression to metastatic)
- Death due to any cause
- Cost-effectiveness of screening

Adverse outcome variables to monitor screening-related harms are:

For peer review only

- Overdiagnosis of clinically insignificant prostate cancer
- Quality of life impacts of screening and quality of life among men with PCa (EPIC26 instrument)
- Prostate cancer-related anxiety (MAX-PC questionnaire)
- Complications from biopsy (PRECISION questionnaire)

Statistical analysis

The main analyses will rely on the intention to screen (ITS) principle and will include all randomized men in the two trial arms who were alive and eligible (free of prostate cancer) at the date of randomization. Those men who became ineligible between the date of randomization and first screening invitation will remain in the ITS analysis set. Men who were ineligible at the time of randomization, but recognized as such only after randomization, will be excluded from the ITS analysis set.

Two-sided statistical tests will be used, and the overall significance level will be set at 5%. Corresponding p-values will be accompanied with estimates of differences and their 95% confidence intervals.

Analysis of the primary outcome

The primary outcome of the trial is death from prostate cancer. This is a superiority trial regarding the primary outcome and the comparisons between trial arms will be analyzed and presented on this basis.

Those men who survived will be considered as right-censored observations at the time passed between the time of analysis and time of randomization period. Those men who were lost to follow-up (e.g., due to emigration) will be considered as censored at that particular time (e.g., at emigration). Time to death, defined as the difference between the date of death and date of randomization, will be used as the event time for the analysis.

To evaluate differences between screening and control arms in prostate cancer specific mortality, Gray's test [19] for testing the null hypothesis of equality of cumulative incidence functions will be used. This test differs from the commonly used logrank test in how competing risks of death are treated and is based on the subdistribution hazard of prostate cancer cause of death.

The test will be complemented by reporting the number of PCa deaths, number of men at risk and estimated cumulative incidence functions for each trial arm over follow-up time. The arms will be compared in absolute risks (number needed to invite i.e., the inverse of the risk difference and number needed to diagnose per averted prostate cancer death, i.e., the ratio of excess incidence to mortality reduction), as well as and relative measures of effect (hazard ratios). Descriptive summaries will also be presented by trial centers, age group at randomization.

<u>Secondary analyses of the primary outcome</u>

Fine-Gray model for the subdistribution hazard will be used to conduct analyses adjusted for background factors for the ITS analysis set. Outcomes will be compared between age groups and trial centers, and in case of differences, analyses to control for trial center and for age at randomization (categorized as 50–54, 55–59, 60–65 years) will be conducted.

For peer review only

Per protocol analyses excluding men with repeated non-attendance will be conducted. We will estimate the screening effect on PCa mortality among those with at least one attended screening round relative to the entire control arm, as well as among those with at least two attended screening rounds. Additional analyses to correct for contamination and non-compliance, *i.e.*, estimation of efficacy of screening under ideal circumstances, will be conducted by the method of Cuzick *et al.* [20].

Descriptive analyses to assess effect heterogeneity by center, age group, education and socioeconomic position will be performed to complement per protocol analyses. Additional analyses requested by external reviewers or editors in peer-review processes will also be done.

Analysis of secondary outcomes

Diagnosis of prostate cancer

The analysis of cumulative incidence of PCa by disease aggressiveness intends to assess screening impact on detection of clinically significant PCa (representing potential benefit through early treatment) and clinically insignificant PCa (indicating overdiagnosis). The intention is to assess the extent of detection of clinically significant PCa by screening relative to the control arm, and extent of overdiagnosis relative to the control arm. This will inform about the degree of accomplishing rationale of the trial, i.e., detection of aggressive cases at least similar to that in PSA-based screening, while substantially decreasing the yield of low-risk cases. As screening advances the time of diagnosis by several years (lead time), cumulative incidence will be used as the indicator of risk.

Disease aggressiveness will be defined by the International Society for Urological Pathology (ISUP) Gleason grade group. The analyses will be conducted separately for the detection of clinically significant (Gleason 7+ or ISUP 2+) and clinically insignificant (Gleason <7 or ISUP 1) PCa. In secondary analyses, alternative criteria for csPCa will also be employed including ISUP 3+ (Gleason 4+3 or higher), maximum length of cancer tissue in biopsy and number of biopsy cores with cancer.

Risk differences and ratios will be used to infer screening benefits and overdiagnosis compared to the control arm. Besides cumulative incidence, the ratio of aggressive to non-aggressive cases (or proportion of aggressive cancers out of all PCa) will also be reported.

Cumulative incidence for both outcomes will be estimated by trial arm. The overall PCa incidence combines screening benefits and harms and is thus regarded of minor importance in the interpretation of screening impact. Tabular presentations of age at diagnosis, disease stage and grade at diagnosis will be presented.

Both intention to screen (by allocation) and per protocol (screening participants and non-participants) analyses will be conducted for each screening round. For screening participants, screen-detected and interval cases will be reported separately, and screen-detected cases will be broken down by those detected in targeted biopsies of MRI-positive lesions (screening protocol evaluated) and systematic biopsies in screen-negative men with PSA density >0.15 (safety measure to avoid missing clinically significant cases). Any cases detected in a random sample of screen-negative men invited to MRI (analyses to assess underlying prevalence of prostate cancer) will also be reported separately. Analyses to evaluate an optimized screening algorithm will include exclusion of cases with PI-RADS score 3 and kallikrein score calculated also incorporating information on previous biopsies (ignored in the main analysis), as well as using higher cut-off values for PSA and the kallikrein score.

Advanced prostate cancer

For peer review only

The analysis of advanced prostate cancer will compare the cumulative incidence of cancer progression, including metastasis and/or biochemical relapse developing after diagnosis and primary treatment, between the screening and control arms. The purpose of the analysis is to evaluate differences between the arms in the risk of developing a potentially lethal, advanced PCa.

The origin of the analysis will be the time of randomization. Cumulative incidence rates will be estimated by the Kaplan-Meier method, and differences between trial arms will be estimated by Cox regression models adjusted by age at diagnosis.

Death due to any cause

The analysis of all-cause mortality aims to show that the trials arms are comparable with each other and the general male population in Finland. These analyses will not inform about the effectiveness of screening. Cumulative survival and mortality rates will be estimated by the Kaplan-Meier method, from time of randomization, displayed with frequencies of events and men at risk by trial arm, and by age at randomization.

This analysis will focus on the intention to screen analysis set.

Cost-effectiveness

A cost-effectiveness analysis will be performed, incorporating cost data for both out-of-pocket estimated from surveys and service cost data collected from health care providers, as well mortality results (ITS analysis) and utilities based on repeated surveys with 15D and EQ5D instruments (on a random sample of participants). The comparator is no active screening, here represented by the control arm. The main outcome is the incremental cost-effectiveness ratio in terms of costs per quality-adjusted life-year.

A preliminary and exploratory cost effectiveness study can be conducted after the last of the follow-up surveys have been returned, approximately at 3 years after the randomization of the last man into the trial. We plan to undertake a full cost-effectiveness analysis around the time when the evidence on the effectiveness of screening regarding primary outcome has been obtained; this will most likely be near to the analysis at 15 years.

Quality of life

These analyses aim to evaluate the short-term and long-term impacts of screening on generic quality of life as well as disease-specific quality of life among men with PCa. Two disease-specific questionnaires, EPIC26 instrument and MAX-PC questionnaires will be used to measure quality of life at 0, 6, 12, and 24 months from PCa diagnosis in both trial arms.

Standard scoring of the EPIC26 instrument will be used. Summary statistics of the five key domains over time and by trial arm will be calculated to assess changes in quality of life of men with PCa from diagnosis onwards. Summary and domain-grouped scores will be analyzed using applications of linear models (or their nonparametric counterparts, if needed) for repeated measures to evaluate differences in quality of life between the arms following PCa diagnosis. Analyses will be adjusted by age group and disease aggressiveness (but not by stage, which is assumed to mediate the effect of screening on QoL through stage shift).

Prostate cancer related anxiety is measured with the Memorial Anxiety Scale for Prostate Cancer (MAX-PC) questionnaire [21]. Results will be presented as frequencies and percentages for total and subscale scores by trial arm.

For peer review only

Generic quality of life and utilities are evaluated using the 15D and EQ5D instruments as described in the cost-effectiveness section.

Analysis of adverse outcomes

In addition to detection of low-risk disease by screening as an indicator of overdiagnosis, adverse outcomes mainly relate to the harms due to biopsies. Adverse effects of prostate biopsy are monitored using the questionnaire developed for the PRECISION trial covering pain and other symptoms immediately after biopsy and at 30 days following biopsy. The number of biopsies, as well as the number (%) and type of complications among those with biopsies will be reported.

Interim analyses and data monitoring

The first analysis of PCa mortality will be conducted at 10 years and the final analysis at 15 years (i.e. at median follow-up time of 10 or 15 years). As we do not intend to stop the trial at 10 years, these interim analyses will be considered as preliminary information. Interim analyses at 10 years will include also analyses of shorter-term benefits.

To control the overall type I error rate (5%) of the trial, we will employ the O'Brien-Fleming rule for alpha spending function. We set the amount of information at 0.5 at 10 years based on the expected numbers of PCa deaths. Thus, by implementation of the O'Brien-Fleming algorithm, the resulting significance level at 10-year interim analysis will be 0.0056, and at the 15-year final analysis 0.0444.

The analyses of secondary outcomes will not be used to infer about the overall effectiveness of screening. We will consider the analyses of these distinct process measures as individual tests rather than part of disjunction testing, in which case precise interpretation but not multiplicity adjustment will be necessary [22, 23].

Analyses of secondary endpoints informing about the intermediate outcomes of process indicators including participation, cancer detection, validity and diagnostic performance of the tests in the entire population and subgroups (screened men, non-participants, men in the control arm) will be carried out at regular intervals, as sufficient data become available for evaluation. These will inform about potential need to modify the procedures. Side studies using the samples collected will be carried out to identify new indicators of prostate cancer risk and prognosis.

An independent data monitoring committee (DMC) oversees the trial conduct, and its main task is to ensure safety of the participants. Safety in this context means that screening or screening procedures should not lead to unacceptable disadvantage for the participants in the light of screening benefits. This could take place if the screening intervention had materially worse performance in detecting clinically relevant prostate cancer than anticipated, or substantially higher level of overdiagnosis. The DMC is given a report of the screening results initially every six months and after the first year every 12 months. The DMC can also request any additional information they regard as pertinent to their task. In case of concern, the DMC can recommend discontinuation of the trial; in practice that would mean stopping recruitment and discontinuation of further screening procedures. In addition, they have a mandate to suggest modifications to the trial protocol.

Handling of missing data

Extent of missing data will be described, for example, by presenting the number of individuals with missing values per variable.

For peer review only

For outcome variables relying on dates – dates of randomization, censoring, diagnosis or death – incomplete dates will be imputed by 15 (in the case that the day variable was missing, but known month and year), and by 30/6 (in the case that only the year was known).

In case a substantial proportion of men (at least 5%) have missing data on one or more variable needed for the effectiveness analysis in question, multiple imputation methods will be used to demonstrate the robustness of findings [24]. Imputation models will include outcome variables and trial arm in addition to all variables relevant to the particular analysis. Final estimates will be derived by combining estimates and their standard errors across data sets using Rubin's rules.

Data management and quality assurance

RedCap database application is used for data management in the trial, covering all major data types from questionnaires and lab results to MRI findings, diagnoses and causes of death. RedCap allows access defined by two-factor authentication (2FA) and flexible definition of user-specific functions and rights.

In REDCap, variable specific parameters and predetermined options are used to prevent entering invalid data (e.g. predefined values and acceptable ranges). All data is verified from the original data source and monitored monthly. Until the verification, data is saved as incomplete or unverified. Lead times between screening tests are monitored every 6-8 weeks. For the laboratory work (including sampling, processing, and storing) each task has a protocol shared by the study centers. Any deviations from the sample specific protocol are documented.

References

1. Culp MB, Soerjomataram I, Efstathiou JA, *et al.* Recent global patterns in prostate cancer incidence and mortality rates. *Eur Urol* 2020; 77:38-52. PMID: 31493960.
2. Hugosson J, Roobol MJ, Månsson M, *et al.* 16-year follow-up of the European Randomised Study of Screening for Prostate Cancer (ERSPC). *Eur Urol* 2019; 76:43-51. PMID: 30824296.
3. Pinsky PF, Miller E, Prorok P, *et al.* Extended follow-up for prostate cancer incidence and mortality among participants in the PLCO screening trial. *BJU Int* 2019;123:854-860. PMID: 30288918.
4. Ilic D, Djulbegovic M, Jung JH, *et al.* Prostate cancer screening with prostate-specific antigen (PSA) test: a systematic review and meta-analysis. *BMJ* 2018; 362:k3519. doi: 10.1136/bmj.k3519. PMID: 30185521.
5. Fenton JJ, Weyrich MS, Durbin S, *et al.* Prostate-specific antigen-based screening for prostate cancer. *JAMA* 2018; 319:1914-31. PMID: 29801018.
6. Paschen U, Sturz S, Fleer D, *et al.* Assessment of prostate-specific antigen screening. *BJU Int* 2022;129:280-289. PMID: 33961337.
7. Schoots IG, Roobol MJ, Nieboer D, *et al.* Magnetic resonance imaging-targeted biopsy may enhance the diagnostic accuracy of significant prostate cancer detection compared to standard transrectal ultrasound-guided biopsy: a systematic review and meta-analysis. *Eur Urol* 2015; 68:438-50. PMID: 25480312.
8. Ahmed HU, El-Shater Bosaily A, Brown LC, *et al.* Diagnostic accuracy of multi-parametric MRI and TRUS biopsy in prostate cancer (PROMIS): a paired validating confirmatory study. *Lancet* 2017; 389:815-822. PMID: 28110982.

For peer review only

9. Hugosson J, Månsson M, Wallström J, *et al.* Prostate Cancer Screening with PSA and MRI Followed by Targeted Biopsy Only. *N Engl J Med*. 2022; 387:2126-2137. PMID: 36477032.
10. Eklund M, Jäderling F, Discacciati A, *et al*. MRI-Targeted or Standard Biopsy in Prostate Cancer Screening. *N Engl J Med* 2021; 385:908-920. PMID: 34237810.
11. Gamble C, Krishan A, Stocken D, *et al*.. Guidelines for the content of statistical analysis plans in clinical trials. *JAMA* 2017; 318:2337–43. https://doi.org/10.1001/jama.2017.18556.
12. Auvinen A, Rannikko A, Taari K, *et al*. A randomized trial of early detection of clinically significant prostate cancer (ProScreen): study design and rationale. *Eur J Epidemiol* 2017; 32:521-527. PMID: 28762124.
13. Statistics Finland's StatFin online service. Available at: https://statfin.stat.fi/PXWeb/pxweb/en/StatFin/ (Accessed 9 June 2021).
14. Kasivisvanathan V, Stabile A, Neves JB, *et al*. Magnetic Resonance Imaging-targeted Biopsy Versus Systematic Biopsy in the Detection of Prostate Cancer: A Systematic Review and Meta-analysis. Eur Urol. 2019;76:284-303. PMID: 31130434.
15. Schoenfeld DA. Sample-size formula for the proportional-hazards regression model. *Biometrics* 1983; 39:499-503. PMID: 6354290.
16. de Rooij M, Israël B, Tummers M, *et al.* ESUR/ESUI consensus statements on multi-parametric MRI for the detection of clinically significant prostate cancer: quality requirements for image acquisition, interpretation and radiologists' training. *Eur Radiol* 2020; 30:5404-5416. PMID: 32424596.
17. Mäkinen T, Karhunen P, Aro J, *et al.* Assessment of causes of death in a prostate cancer screening trial. *Int J Cancer* 2008; 122: 413-417. https://doi.org/10.1002/ijc.23126
18. Kilpeläinen TP, Mäkinen T, Karhunen PJ, *et al.* Estimating bias in causes of death ascertainment in the Finnish Randomized Study of Screening for Prostate Cancer. *Cancer Epidemiol* 2016; 45:1-5. PMID: 27636505.
19. Gray RJ: A class of K-sample tests for comparing the cumulative incidence of a competing risk. *Ann Stat* 1988; 16:1141-1154.
20. Cuzick J, Edwards R, Segnan N. Adjusting for non-compliance and contamination in randomized clinical trials. *Stat Med* 1997; 16:1017-29. doi: 10.1002/(sici)1097-0258(19970515)16:9<1017::aid-sim508>3.0.co;2-v. Erratum in: *Stat Med* 2007;26:3821. PMID: 9160496.
21. Roth AJ, Rosenfeld, B, Kornblith AB, *et al.* The memorial anxiety scale for prostate cancer. *Cancer* 2003; 97:2910-18. PMID: 12767107.
22. Parker RA, Weir CJ. Multiple secondary outcome analyses: precise interpretation is important. *Trials* 2022; **23:** 27. https://doi.org/10.1186/s13063-021-05975-2
23. Rubin M. When to adjust alpha during multiple testing: a consideration of disjunction, conjunction, and individual testing. *Synthese* 2021; **199:** 10969–11000. https://doi.org/10.1007/s11229-021-03276-4
24. Little RJ, D'Agostino R, Cohen ML, *et al.* The prevention and treatment of missing data in clinical trials. *N Engl J Med* 2012; 367:1355-60. PMID: 23034025.

Authors' contributions

All authors approved the final version of this manuscript. Study concept and design: AA, AR, JN, JR, KN, TK, TT; drafting of the manuscript: AA, JN; critical revision of the manuscript for important intellectual content: AA, AR, JN, JR, KN, TK, TT.

For peer review only - http://bmjopen.bmj.com/site/about/guidelines.xhtml

Funding statement

This work was supported by the Academy of Finland (grant number 311336) the Finnish Cancer Foundation, the Jane and Aatos Erkko foundation, Competitive State Research Funding administered by Tampere University Hospital (grant number 9V02), and Päivikki and Sakari Sohlberg Foundation.

Competing interest statement

Antti Rannikko declares receipt of lecture and consultation fees from Janssen and Orion.

For peer review only

SPIRIT
STANDARD PROTOCOL ITEMS: RECOMMENDATIONS FOR INTERVENTIONAL TRIALS

SPIRIT 2013 Checklist: Recommended items to address in a clinical trial protocol and related documents*

Section/item	Item No	Description	Addressed on page number
**Administrative information**			
Title	1	Descriptive title identifying the study design, population, interventions, and, if applicable, trial acronym	___1_________
Trial registration	2a	Trial identifier and registry name. If not yet registered, name of intended registry	___1_________
	2b	All items from the World Health Organization Trial Registration Data Set	___N/A_______
Protocol version	3	Date and version identifier	____3________
Funding	4	Sources and types of financial, material, and other support	____14______
Roles and responsibilities	5a	Names, affiliations, and roles of protocol contributors	____1_______
	5b	Name and contact information for the trial sponsor	____N/A______
	5c	Role of study sponsor and funders, if any, in study design; collection, management, analysis, and interpretation of data; writing of the report; and the decision to submit the report for publication, including whether they will have ultimate authority over any of these activities	____N/A______
	5d	Composition, roles, and responsibilities of the coordinating centre, steering committee, endpoint adjudication committee, data management team, and other individuals or groups overseeing the trial, if applicable (see Item 21a for data monitoring committee)	____N/A______

For peer review only

Introduction

Background and rationale	6a	Description of research question and justification for undertaking the trial, including summary of relevant studies (published and unpublished) examining benefits and harms for each intervention	__3__________
	6b	Explanation for choice of comparators	__NA________
Objectives	7	Specific objectives or hypotheses	__1__________
Trial design	8	Description of trial design including type of trial (eg, parallel group, crossover, factorial, single group), allocation ratio, and framework (eg, superiority, equivalence, noninferiority, exploratory)	__3,8________

Methods: Participants, interventions, and outcomes

Study setting	9	Description of study settings (eg, community clinic, academic hospital) and list of countries where data will be collected. Reference to where list of study sites can be obtained	__N/A_______
Eligibility criteria	10	Inclusion and exclusion criteria for participants. If applicable, eligibility criteria for study centres and individuals who will perform the interventions (eg, surgeons, psychotherapists)	___3-4_______
Interventions	11a	Interventions for each group with sufficient detail to allow replication, including how and when they will be administered	___4-5_______
	11b	Criteria for discontinuing or modifying allocated interventions for a given trial participant (eg, drug dose change in response to harms, participant request, or improving/worsening disease)	___N/A_______
	11c	Strategies to improve adherence to intervention protocols, and any procedures for monitoring adherence (eg, drug tablet return, laboratory tests)	___N/A_______
	11d	Relevant concomitant care and interventions that are permitted or prohibited during the trial	___N/A_______
Outcomes	12	Primary, secondary, and other outcomes, including the specific measurement variable (eg, systolic blood pressure), analysis metric (eg, change from baseline, final value, time to event), method of aggregation (eg, median, proportion), and time point for each outcome. Explanation of the clinical relevance of chosen efficacy and harm outcomes is strongly recommended	___7-8_______
Participant timeline	13	Time schedule of enrolment, interventions (including any run-ins and washouts), assessments, and visits for participants. A schematic diagram is highly recommended (see Figure)	___4-5________

For peer review only - http://bmjopen.bmj.com/site/about/guidelines.xhtml

Sample size	14	Estimated number of participants needed to achieve study objectives and how it was determined, including clinical and statistical assumptions supporting any sample size calculations	___4_________
Recruitment	15	Strategies for achieving adequate participant enrolment to reach target sample size	___4_________

Methods: Assignment of interventions (for controlled trials)

Allocation:			
Sequence generation	16a	Method of generating the allocation sequence (eg, computer-generated random numbers), and list of any factors for stratification. To reduce predictability of a random sequence, details of any planned restriction (eg, blocking) should be provided in a separate document that is unavailable to those who enrol participants or assign interventions	___4-5_______
Allocation concealment mechanism	16b	Mechanism of implementing the allocation sequence (eg, central telephone; sequentially numbered, opaque, sealed envelopes), describing any steps to conceal the sequence until interventions are assigned	___5_________
Implementation	16c	Who will generate the allocation sequence, who will enrol participants, and who will assign participants to interventions	___5_________
Blinding (masking)	17a	Who will be blinded after assignment to interventions (eg, trial participants, care providers, outcome assessors, data analysts), and how	___N/A_______
	17b	If blinded, circumstances under which unblinding is permissible, and procedure for revealing a participant's allocated intervention during the trial	___N/A_______

Methods: Data collection, management, and analysis

Data collection methods	18a	Plans for assessment and collection of outcome, baseline, and other trial data, including any related processes to promote data quality (eg, duplicate measurements, training of assessors) and a description of study instruments (eg, questionnaires, laboratory tests) along with their reliability and validity, if known. Reference to where data collection forms can be found, if not in the protocol	__7,12_______
	18b	Plans to promote participant retention and complete follow-up, including list of any outcome data to be collected for participants who discontinue or deviate from intervention protocols	___N/A_______

Data management	19	Plans for data entry, coding, security, and storage, including any related processes to promote data quality (eg, double data entry; range checks for data values). Reference to where details of data management procedures can be found, if not in the protocol	___12________
Statistical methods	20a	Statistical methods for analysing primary and secondary outcomes. Reference to where other details of the statistical analysis plan can be found, if not in the protocol	____7-11_____
	20b	Methods for any additional analyses (eg, subgroup and adjusted analyses)	____9-11_____
	20c	Definition of analysis population relating to protocol non-adherence (eg, as randomised analysis), and any statistical methods to handle missing data (eg, multiple imputation)	___8, 11-12___
**Methods: Monitoring**			
Data monitoring	21a	Composition of data monitoring committee (DMC); summary of its role and reporting structure; statement of whether it is independent from the sponsor and competing interests; and reference to where further details about its charter can be found, if not in the protocol. Alternatively, an explanation of why a DMC is not needed	____11______
	21b	Description of any interim analyses and stopping guidelines, including who will have access to these interim results and make the final decision to terminate the trial	____11______
Harms	22	Plans for collecting, assessing, reporting, and managing solicited and spontaneously reported adverse events and other unintended effects of trial interventions or trial conduct	____11_______
Auditing	23	Frequency and procedures for auditing trial conduct, if any, and whether the process will be independent from investigators and the sponsor	____N/A______
**Ethics and dissemination**			
Research ethics approval	24	Plans for seeking research ethics committee/institutional review board (REC/IRB) approval	_____1,3_____
Protocol amendments	25	Plans for communicating important protocol modifications (eg, changes to eligibility criteria, outcomes, analyses) to relevant parties (eg, investigators, REC/IRBs, trial participants, trial registries, journals, regulators)	___N/A______

For peer review only - http://bmjopen.bmj.com/site/about/guidelines.xhtml

Consent or assent	26a	Who will obtain informed consent or assent from potential trial participants or authorised surrogates, and how (see Item 32)	__N/A________
	26b	Additional consent provisions for collection and use of participant data and biological specimens in ancillary studies, if applicable	__N/A________
Confidentiality	27	How personal information about potential and enrolled participants will be collected, shared, and maintained in order to protect confidentiality before, during, and after the trial	__N/A________
Declaration of interests	28	Financial and other competing interests for principal investigators for the overall trial and each study site	__N/A________
Access to data	29	Statement of who will have access to the final trial dataset, and disclosure of contractual agreements that limit such access for investigators	__N/A________
Ancillary and post-trial care	30	Provisions, if any, for ancillary and post-trial care, and for compensation to those who suffer harm from trial participation	__N/A________
Dissemination policy	31a	Plans for investigators and sponsor to communicate trial results to participants, healthcare professionals, the public, and other relevant groups (eg, via publication, reporting in results databases, or other data sharing arrangements), including any publication restrictions	__3__________
	31b	Authorship eligibility guidelines and any intended use of professional writers	__N/A________
	31c	Plans, if any, for granting public access to the full protocol, participant-level dataset, and statistical code	__N/A________
**Appendices**			
Informed consent materials	32	Model consent form and other related documentation given to participants and authorised surrogates	___attached___
Biological specimens	33	Plans for collection, laboratory evaluation, and storage of biological specimens for genetic or molecular analysis in the current trial and for future use in ancillary studies, if applicable	___N/A_______

*It is strongly recommended that this checklist be read in conjunction with the SPIRIT 2013 Explanation & Elaboration for important clarification on the items. Amendments to the protocol should be tracked and dated. The SPIRIT checklist is copyrighted by the SPIRIT Group under the Creative Commons "Attribution-NonCommercial-NoDerivs 3.0 Unported" license.

For peer review only - http://bmjopen.bmj.com/site/about/guidelines.xhtml
